# An Outline of the Latest Crystallographic Studies on Inhibitor-Enzyme Complexes for the Design and Development of New Therapeutics against Tuberculosis

**DOI:** 10.3390/molecules26237082

**Published:** 2021-11-23

**Authors:** Matteo Mori, Stefania Villa, Samuele Ciceri, Diego Colombo, Patrizia Ferraboschi, Fiorella Meneghetti

**Affiliations:** 1 Department of Pharmaceutical Sciences, University of Milan, Via L. Mangiagalli 25, 20133 Milano, Italy; matteo.mori@unimi.it (M.M.); stefania.villa@unimi.it (S.V.); fiorella.meneghetti@unimi.it (F.M.); 2 Department of Medical Biotechnology and Translational Medicine, University of Milan, Via C. Saldini 50, 20133 Milano, Italy; diego.colombo@unimi.it (D.C.); patrizia.ferraboschi@unimi.it (P.F.)

**Keywords:** tuberculosis, structure-based drug design, fragment-based drug design, *Mycobacterium tuberculosis*, structure–activity relationships (SARs)

## Abstract

The elucidation of the structure of enzymes and their complexes with ligands continues to provide invaluable insights for the development of drugs against many diseases, including bacterial infections. After nearly three decades since the World Health Organization’s (WHO) declaration of tuberculosis (TB) as a global health emergency, *Mycobacterium tuberculosis* (*Mtb*) continues to claim millions of lives, remaining among the leading causes of death worldwide. In the last years, several efforts have been devoted to shortening and improving treatment outcomes, and to overcoming the increasing resistance phenomenon. The structural elucidation of enzyme-ligand complexes is fundamental to identify hot-spots, define possible interaction sites, and elaborate strategies to develop optimized molecules with high affinity. This review offers a critical and comprehensive overview of the most recent structural information on traditional and emerging mycobacterial enzymatic targets. A selection of more than twenty enzymes is here discussed, with a special emphasis on the analysis of their binding sites, the definition of the structure–activity relationships (SARs) of their inhibitors, and the study of their main intermolecular interactions. This work corroborates the potential of structural studies, substantiating their relevance in future anti-mycobacterial drug discovery and development efforts.

## 1. Introduction

*Mycobacterium tuberculosis* (*Mtb*), the causative agent of tuberculosis (TB), is the most devastating human pathogen, as confirmed by the latest TB Report published in October [1]. The protracted antibiotic therapy, even if cheap and with high cure rates among treated patients, presents a number of negative factors and is hindered by many issues, including the phenomenon of persistence, the drug resistance, and the co-infection with HIV. Once inhaled into the lungs, the bacterium is phagocytosed by macrophages, where it encounters an environment limited in many nutrients, such as sugars and amino acids. Under these nutritionally limited conditions, enzymes essential for the growth and the survival of *Mtb* represent attractive targets for the design of new potential drugs.

Considering the increasing difficulty of modern-day drugs to effectively tackle TB, many researchers are directing their efforts toward the discovery of new medicines.

Understanding enzyme inhibition processes can help conceptualize different types of inhibitors and characterize potential anti-mycobacterial pathways/mechanisms. Exploiting the structural information allows the thorough evaluation of diverse compounds, providing the knowledge required to efficiently optimize leads toward differentiated candidate drugs.

Here, we report an update on the latest structural studies (2018–present) performed to elucidate the inhibition mechanisms of novel antitubercular agents against validated *Mtb* target enzymes. Our work complements previous overviews of crystallographic structures relevant for anti-TB drug discovery [2,3].

This compendium will hopefully prove useful in providing inspiration for the design and development of new antitubercular agents, highlighting valuable data to be combined with complementary medicinal chemistry approaches.

## 2. Mtb Enzymatic Targets

A selection of structural studies (2018–present) on chemical entities exhibiting anti-TB properties and acting on specific *Mtb* enzymes is here reported with the intent to highlight the most promising candidates and their key contacts within the binding site of the target. The understanding of the ligand-enzyme interaction is fundamental to rationalize medicinal chemistry efforts in the discovery and development of new drugs, to predict potential off-target interactions with human counterparts, and to evaluate the likelihood of possible transfers of resistance factors among bacteria.

### 2.1. Isocitrate Lyase 1 (ICL1)

**Scheme 1:**
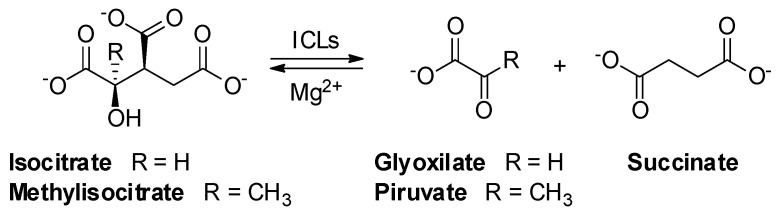
Reversible lysis reaction catalyzed by ICLs in the glyoxylate shunt: conversion of isocitrate to glyoxylate and succinate.

Fatty acids have been shown to be a major source of carbon and energy for *Mtb* in clinically infected lungs. Two catabolic pathways have been described for their utilization: the β-oxidation cycle and the glyoxylate shunt. The glyoxylate shunt circumvents the loss of two carbon dioxides from the tricarboxylic acid (TCA) cycle, thereby permitting the net incorporation of carbon into cellular structures during growth on acetate. This mechanism is widespread among prokaryotes, lower eukaryotes, and plants but it is absent in vertebrates. The glyoxylate shunt is particularly important for the persistence of various infectious agents, including *Mtb*. Interestingly, an upregulation of isocitrate lyases (ICL, isoform 1 and 2) has been observed in bacteria and fungi during infection [4]. ICLs are key enzymes converting isocitrate/methylisocitrate to glyoxylate/pyruvate and succinate in the glyoxylate shunt and methylcitrate cycle, respectively (Scheme 1) [5]. Hence, because ICLs play a crucial role in both persistence and virulence, they can be considered ideal targets for the development of new anti-TB agents [4].

**Figure 1:**
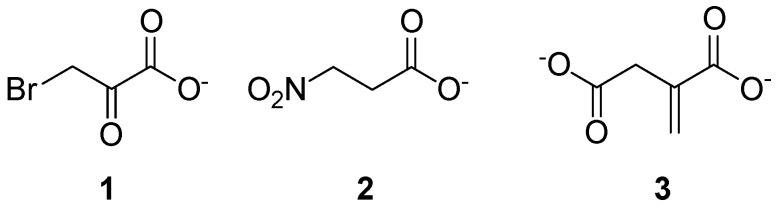
Chemical structure of compounds **1**–**3**.

For this purpose, the 3D structures of *Mtb* ICL1 in the apo form (PDB: 1F61) and in complex with the inhibitors 3-bromopyruvate (**1**), 3-nitropropionate (**2**), and itaconate (**3**) (Figure 1) were studied by means of X-ray diffraction (XRD). In the host, **3** is synthesized by decarbonylation of *cis*-aconitate upon activation of immune cells [6]. Despite the enzyme acts as a tetramer, it is also stable in solution as a dimer. The monomeric structure of ICL1 consists of sixteen α-helices, five 3_10_-helices, twelve β-strands, and several loops; the active sites are located near the interface of the two subunits.

**Figure 2:**
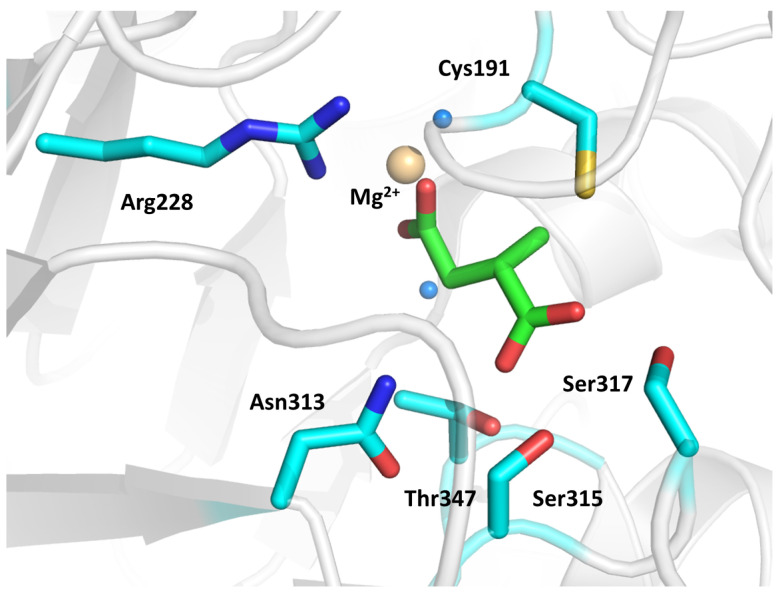
Crystal structure of **3** in complex with ICL1 (PDB: 6XPP). The ligand and interacting residues are represented as green and cyan sticks, respectively. The Mg^2+^ ion is shown as a golden sphere, while ordered water molecules are visualized as blue spheres.

The inhibition mechanism of **1** is based on a dehalogenation, followed by the formation of a covalent adduct with the active site nucleophile Cys191; supposedly, this modification produces conformational changes in the L5 loop and C-terminal tail, ultimately leading to the closure of the binding region. The most recent structure of ICL1 in complex with **3** (IC_50_ = 420 μM) showed that the inhibitor was bound to the active site of the enzyme (Figure 2, PDB: 6XPP). In detail, the hydrogen bonding interactions between the carboxylate moieties of the inhibitor and Asn313, Ser315, Ser317, Thr347, Arg228, and Mg^2+^ were found to be essential for the binding. Adding hydrophobic, aromatic, and polar moieties to the alkene of **3** significantly lowered the inhibition potency, suggesting that its proximity to Cys191 is a prerequisite for the covalent interaction to occur. The crystal structure of the ICL-**3** complex revealed that the enzyme was in its closed conformation, consistently with previous data. This finding confirmed that the binding of the compound to the active site is crucial to induce conformational changes in regions that control access to the binding pocket [7].

The crystallographic information provided important insights into the mechanism of ICL1, suggesting that the itaconate scaffold can be utilized to develop new selective inhibitors, branching out at the C3 position of **3** to fill the spacious pocket occupied by glyoxylate.

### 2.2. Methylmalonyl-CoA Mutase (MCM)

**Figure 3:**
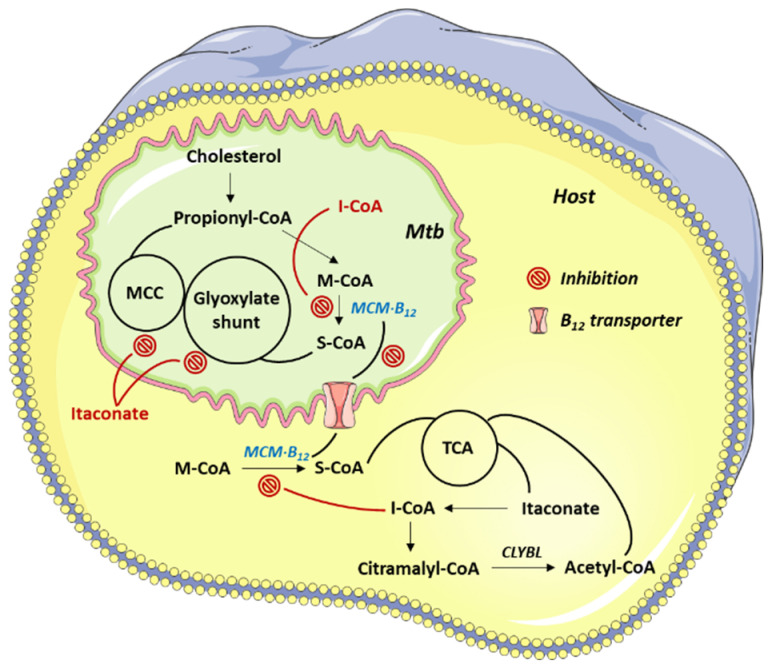
Mtb pathways targeted by **4**.

Mycobacterial B_12_-dependent methylmalonyl-CoA mutase (MCM) catalyzes the reversible isomerization of methylmalonyl-CoA (M-CoA) to succinyl-CoA via a 5′-deoxyadenosylcobalamin (AdoCbl)-dependent radical mechanism (Figure 3).

**Scheme 2:**
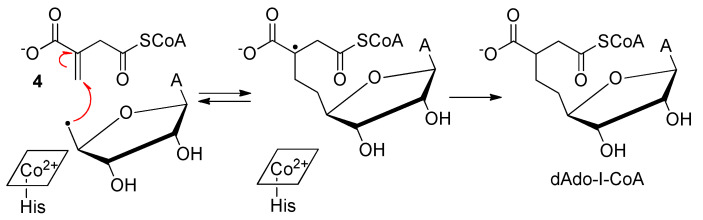
Reaction between dAdo• and **4**.

The coenzyme A (CoA) derivative of **3**, itaconyl-CoA (I-CoA, **4**), is a suicide inactivator of the human and mycobacterial MCMs. Because MCM plays an essential role in lipid breakdown, inhibition studies on this enzyme have recently been reported [8]. The mechanisms underlying **4**-mediated MCM inhibition were studied through the EPR and MS analyses of the reaction products between the 5′-deoxyadenosyl radical (dAdo•) and **4**. The authors concluded that dAdo• added to the double bond of **4** affording a tertiary carbon radical, stabilized by delocalization onto the adjacent carboxylate (Scheme 2).

**Figure 4:**
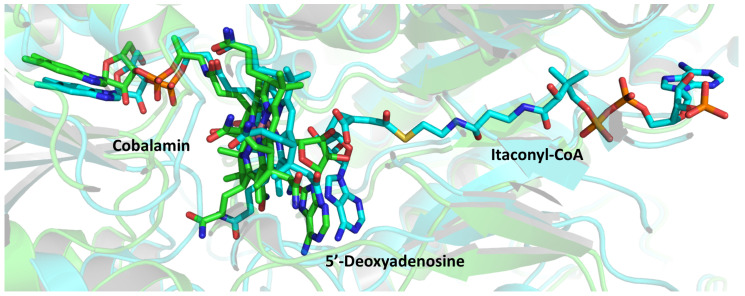
Superimposition of the active sites of native MCM (green, PDB: 6OXC) and of its complex with **4** (cyan, PDB: 6OXD). The ligands are represented as colored sticks.

A crystallographic study of AdoCbl-MCM complexed with **4** allowed to characterize the binding between the enzyme and the inhibitor (Figure 4). In the native heterodimeric structure, AdoCbl inserted its dimethylbenzimidazole tail in a side pocket, while His265 served as the lower axial ligand. On the opposite face, the 5′-carbon of dAdo was 2.5 Å away from the cobalt atom; the adenine was coplanar with the corrin ring and oriented above the pyrrole rings. The binding of **4** induced a large conformational change in the AdoCbl α unit, which collapsed around the binding pocket.

The authors concluded that the homolysis of the cobalt-carbon bond in AdoCbl generated dAdo• and a spectator cobalamin radical; compound **4** triggered this cleavage, mimicking a normal catalytic cycle, but proximity effects promoted suicidal addition of dAdo• to its double bond.

The demonstration that **4** is an inhibitor of MCM is consistent with the reported observation that the incidence of active TB is markedly lower in patients affected by pernicious anemia, which causes B_12_ deficiency [9].

### 2.3. Fumarate Hydratase

**Scheme 3:**
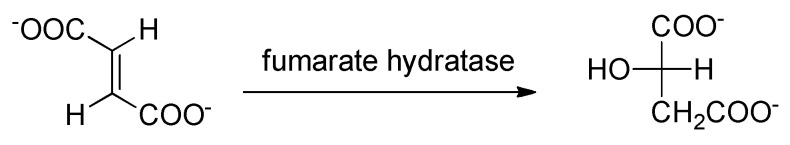
Reversible lysis reaction catalyzed by fumarase.

Fumarate hydratase (fumarase), a component of the citric acid cycle, is essential for *Mtb* survival. This enzyme catalyzes the reversible hydration of fumarate to malate (Scheme 3), and its depletion has been linked to an impaired mycobacterial growth due to the accumulation of fumarate, which reacts with cysteine thiols. The resulting oxidative stress causes the death of the microorganism both in vitro and in vivo. Unfortunately, targeting fumarase poses a significant challenge because the protein is highly evolutionarily conserved. In particular, the human and *Mtb* homologs share identical active site residues, as well as a 53% overall sequence identity.

The mycobacterial enzyme is a 210 kDa class-II fumarase, featuring a homotetrameric structure. Each subunit is characterized by an N-terminal domain (residues 1–137), a central domain (residues 138–393), and a flexible C-terminal domain (residues 394–474). The protein has four active sites, located in a cleft formed by three subunits, and covered by a loop that allows the switch between “open” and “closed” states.

**Figure 5:**
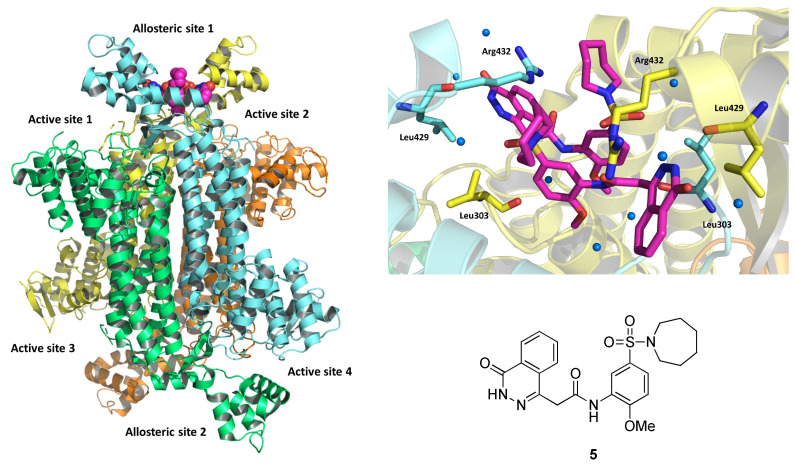
On the **left**, depiction of the X-ray crystal structure of the fumarase-**5** complex (PDB: 5F91). The four chains are evidenced in different colors, and the two bound inhibitors are represented as magenta spheres. On the **top right**, expansion of the binding site, showing the ligand as magenta sticks. Interacting residues are represented as yellow and light-blue sticks depending on the chain to which they belong. On the **bottom right**, chemical structure of **5**.

In 2016, the discovery of an inhibitor, compound **5** (IC_50_ = 2.5 μM), was reported [10]. The selectivity of the molecule was due to its binding to an allosteric pocket, composed of unconserved residues located on two C-terminal domains of the fumarase tetramer (Figure 5).

**Figure 6:**
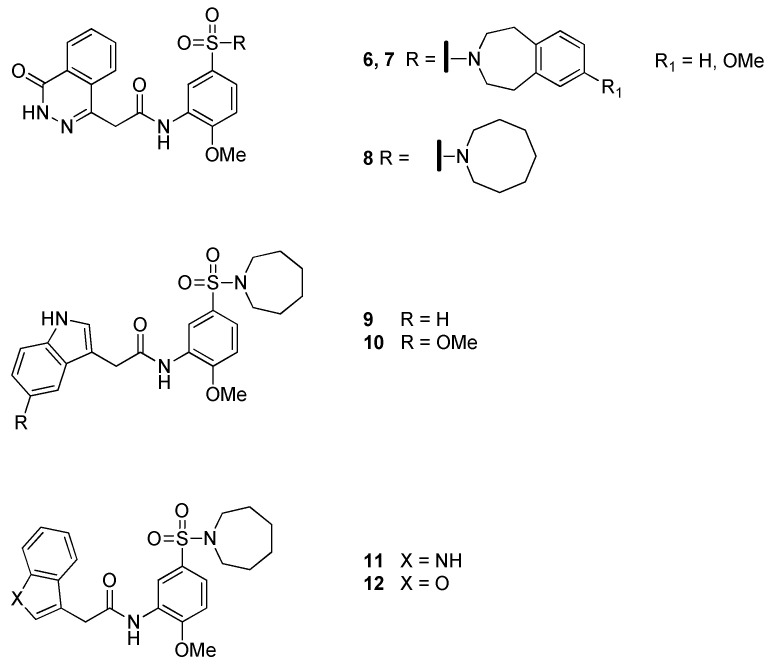
Chemical structure of compounds **6**–**12**.

Despite **5** did not exhibit bactericidal activity, it was able to inhibit the growth of the mycobacterium; hence, additional investigations were carried out in 2019 by the same research group [11]. In detail, **5** was deconstructed into “fragment-like” molecules, affording a library of compounds with a molecular weight in the range 204–326 Da. However, this defragmentation approach proved to be unsuccessful, as evidenced by both biochemical assays and differential scanning fluorimetry (DSF) tests. Therefore, a traditional structure–activity relationship (SAR) study was undertaken. A series of compounds, resulting from modifications of the different moieties of **5**, was prepared and screened against the enzyme. The authors identified two benzoazepanyl derivatives, **6** and **7**, showing a three-fold stronger inhibition compared to **5**, and a set of compounds (**8**–**12**, Figure 6) endowed with a measurable minimal inhibitory concentration (MIC) against *Mtb* in vitro (6.3–9 μM in 7H9/DPPC). All derivatives maintained the dimeric binding mode to the allosteric site of the enzyme, as confirmed by XRD. These encouraging results confirm the importance of fumarase as a target for the development of bacteriostatic compounds against TB.

### 2.4. Enoyl-Acyl Carrier Protein Reductase (InhA)

**Scheme 4:**
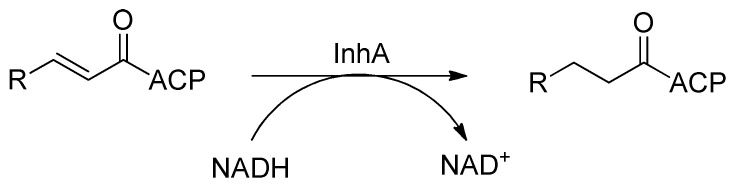
InhA-catalyzed reduction of trans-2-enoyl-ACP.

Enoyl-acyl carrier protein reductase (InhA) catalyzes the reduction of trans-2-enoyl-acyl carrier protein (trans-2-enoyl-ACP) in the mycobacterial fatty acid biosynthetic pathway (Scheme 4). This enzyme is the target of isoniazid, a frontline antitubercular drug, which acts by forming a covalent adduct with NAD.

**Figure 7:**
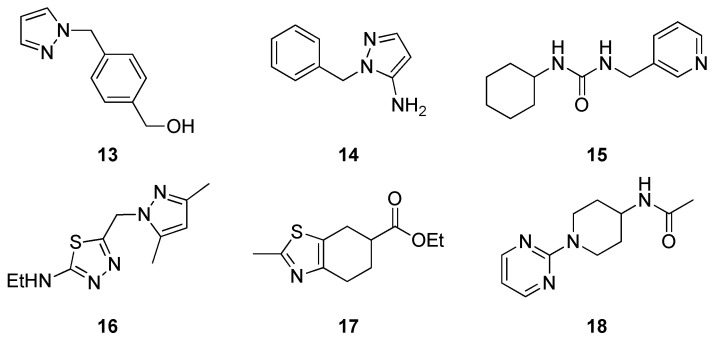
Fragments **13**–**18** from which cocrystal structures were obtained.

The kinetic parameters are important characteristics of a drug. Equilibrium properties (*K_d_* and IC_50_) are usually used to predict the in vivo efficacy, but variations in the concentrations, for example of the inhibitor, must be considered too. Residence time, the reciprocal of the dissociation rate constant, represents the “lifespan” of an enzyme–inhibitor complex. Previous studies [12,13] showed that the active site loop of InhA undergoes an isomerization from a closed state to an open one in the course of the catalytic process, and that the residence time of ligands can be increased by tailoring their interactions during the isomerization event. Different approaches have been employed over the years to identify direct InhA inhibitors. Moreover, the increasing spread of isoniazid-resistant isolates has forced the scientific community to envisage new strategies to bypass these adaptation mechanisms. In this respect, fragment-based drug design (FBDD) has been considered by different research teams. In 2018, Prati and collaborators screened 1360 fragments against the NADH-bound form of InhA, using an ^1^H NMR technique (saturation transfer difference, STD) [14]. The 149 selected fragments were submitted to a biochemical assay and, among the 32 compounds showing a potential activity, 15 were selected for crystallographic studies. Six fragments (Figure 7) afforded crystals suitable for structure determination. The InhA-NADH cocrystal structures of fragments **17** and **18** revealed additional interactions with InhA compared to other fragments [14]. The incorporated functional groups allowed an easy elaboration to afford optimized InhA inhibitors.

**Figure 8:**
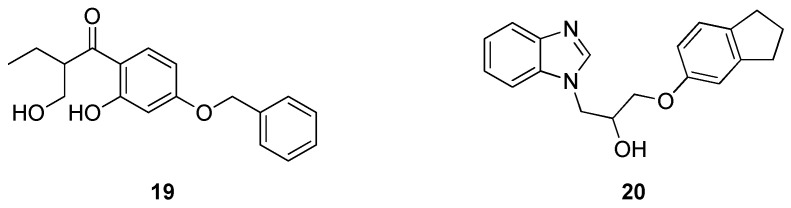
Chemical structure of compounds **19** and **20**.

More recently, a similar approach based on a virtual screening (VS) supported by biological assays was published [15]. Sixteen compounds were selected and subjected to in vitro tests; among them, two compounds, **19** and **20**, proved to be active against *Mtb* (Figure 8).

**Figure 9:**
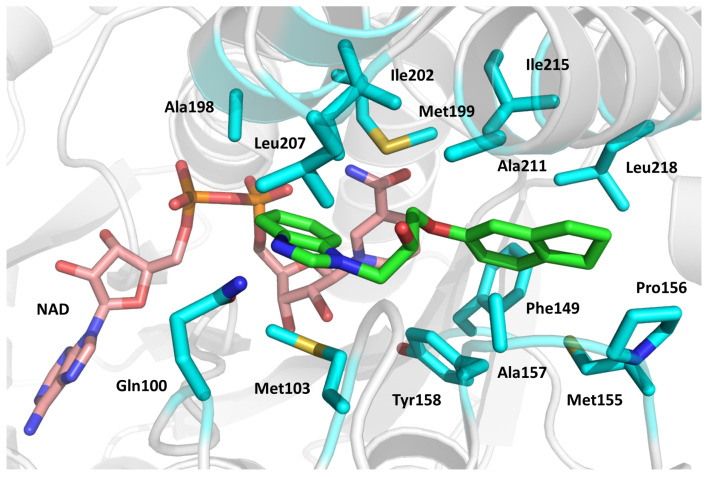
Compound **20** bound to the active site of InhA (PDB: 6R9W). The ligand and interacting residues are represented as green and cyan sticks, respectively. The NAD cofactor is shown as pink sticks.

The binding mode of the most potent compound **20** was investigated by X-ray crystallography (Figure 9).

Compound **20** bound the InhA active site in a hydrophobic pocket, with its hydroxyl group forming a hydrogen bond with the backbone carbonyl oxygen of Ala211. This study also allowed to establish that the side chain of Tyr158 was in the “in” orientation. This conformation was associated with the ternary InhA complex (substrate/cofactor-bound form), whereas the “out” conformation was associated with the binary InhA complex (cofactor-bound form). The inhibitor was located at some distance from the NAD^+^ cofactor, with the benzimidazole group neighboring the NAD^+^ nicotinamide ring and the diphosphate group. Interestingly, the binding mode of **20** was different from that of most of the other inhibitors: it replicated the interaction of the substrate acyl chain at a site distant from the catalytic center and the NAD^+^ binding site. This similarity with the substrate binding justified further studies and optimization.

**Figure 10:**
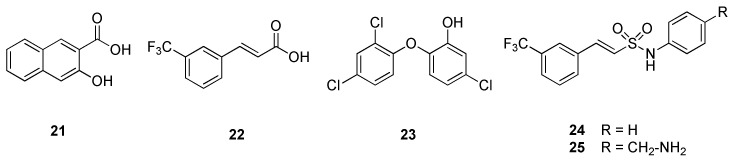
Chemical structure of compounds **21**–**25**.

In 2020, a new FBDD study was undertaken with the aim of finding novel InhA inhibitors: a three-stage screening (thermal shift, ligand-based NMR, and X-ray crystallography) led to the identification of several promising fragment hits (some of them are shown in Figure 10, **21**–**25**) [16].

Starting from a library of 200 molecules, a thermal shift assay was used to test the potential inhibitors in the presence of NAD^+^ and triclosan (positive control). Then, the selected hits were examined by ligand-based NMR techniques. The 18 hits validated by NMR were co-crystallized with InhA, and the X-ray diffraction data were collected. The binding pockets of InhA were classified by these authors into three distinct sites, endowed with different properties: site I, the catalytic site, site II, a hydrophobic region that binds fatty acid chains, and site III, a solvent-exposed site known as the size-limiting region. The X-ray crystal structures included the NAD^+^ cofactor and the compounds, which occupied the same area as the substrate (sites I and II). Interestingly, the crystal structure of InhA in complex with **21** did not show Tyr158 in the usual “in” configuration, probably because the residue was involved in a π-π stacking interaction with the compound, which was sandwiched between it and Phe149 (PDB: 6SQ9).

Overall, the structural information allowed to perform fragment optimization, which led to increased inhibitory activities against InhA. However, the effects on the enzyme were not reflected in high antitubercular activities, suggesting that these compounds had permeability issues or were metabolized.

### 2.5. Dihydrofolate Reductase (DHFR)

**Scheme 5:**
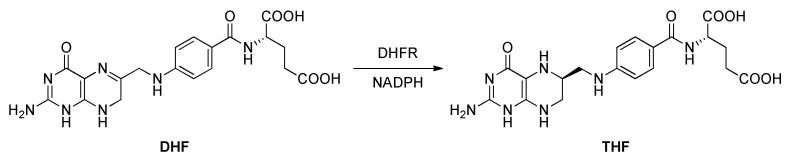
Catalytic activity of the DHFR enzyme.

The folate biosynthetic pathway regulates the production of reduced folate cofactors, essential for the de novo synthesis of methionine purines and deoxythymidine monophosphate (dTMP). The dihydrofolate reductase (DHFR) catalyzes the reduction of dihydrofolate (DHF) to tetrahydrofolate (THF), using NADPH as the reducing agent (Scheme 5).

Current *Mtb* DHFR inhibitors either show poor potency or fail to block the growth of the pathogen, most likely due to a lack of permeability.

**Figure 11:**
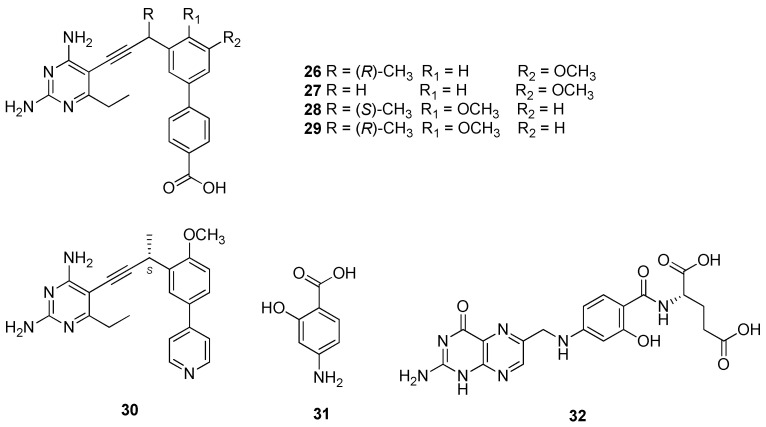
Chemical structure of INCA derivatives **26**–**32**.

In 2019, Hajian and collaborators reported the screening of a class of “ionized non-classical antifolates” (INCAs, Figure 11) versus DHFR [17].

**Figure 12:**
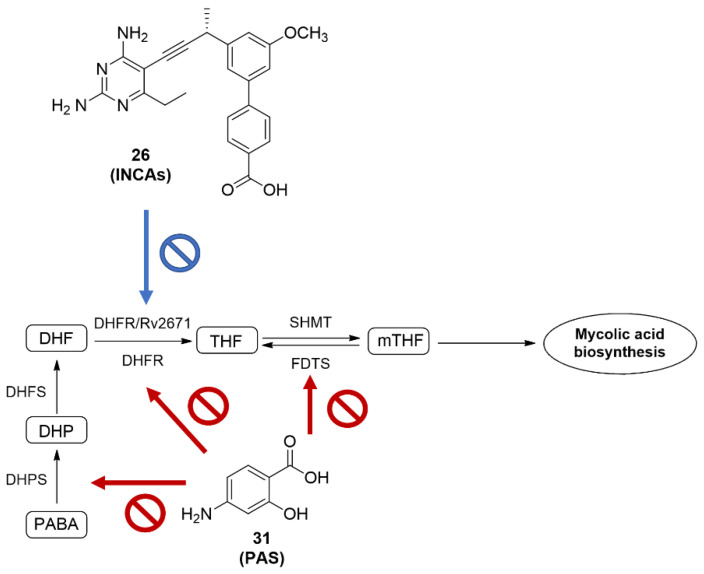
Inhibition mechanisms of INCAs and PAS [17].

The presence of a single acidic functionality made the compounds ionic at all pH values. Differently from lipophilic antifolates or classical antifolates, which carry a double negative charge, INCAs were either zwitterions or featured a single net negative charge (−1). The activity of these molecules was compared to that of *p*-amino salicylic acid (PAS, **31**), the only antifolate currently used in anti-TB therapy (Figure 12).

The effect of INCAs was also investigated on an alternative mycobacterial folate pathway, which produces THF and dTMP by employing two recently identified enzymes: the reductase Rv2671 and a flavin-dependent thymidylate synthase. Notably, the presence of these two enzymes provides the microorganism with a source of resistance to antifolates. INCAs proved to be efficient inhibitors of both DHFR and Rv2671. Moreover, they showed a long-lasting target occupancy, a desirable feature for antibacterial agents. 

**Figure 13:**
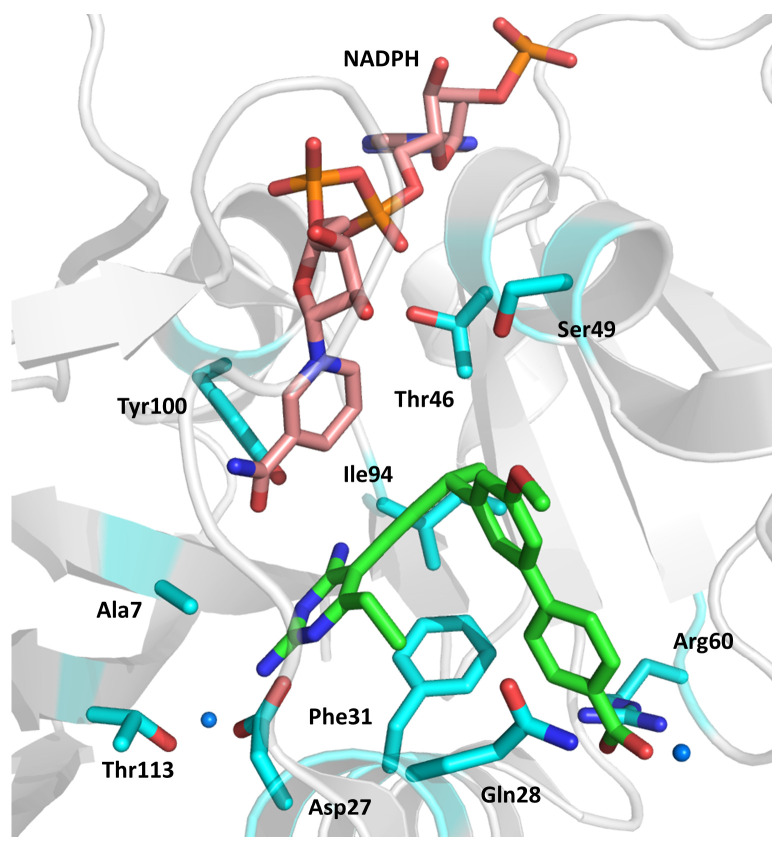
DHFR in complex with NADPH (pink sticks) and **26** (green sticks) at 1.4 Å (PDB: 6DDP). Interacting residues are represented as cyan sticks, while ordered water molecules are shown as blue spheres.

The crystallization of compounds **26** (Figure 13), **27**, and **32** with DHFR allowed to solve the structure of the ternary complex with NADPH. Considering the cocrystal with **26**, the 2,4-diaminopyrimidine ring was positioned into a hydrophobic pocket, making two hydrogen bonds with the side chain of Asp27, and two hydrogen bonds with Ile5 and Ile94. The biaryl system made hydrophobic interactions with Glu28, Leu50, Pro51, Ile54, and Leu57. The carboxylate formed a hydrogen bond with Arg60, a pivotal interaction responsible of the high affinity of the ligand. The lower affinity of **30** was attributed to the replacement of the benzoic acid with a pyridine, while the poor enzymatic activity of **32** was justified by the inverse orientation of the pteridine ring.

**Figure 14:**
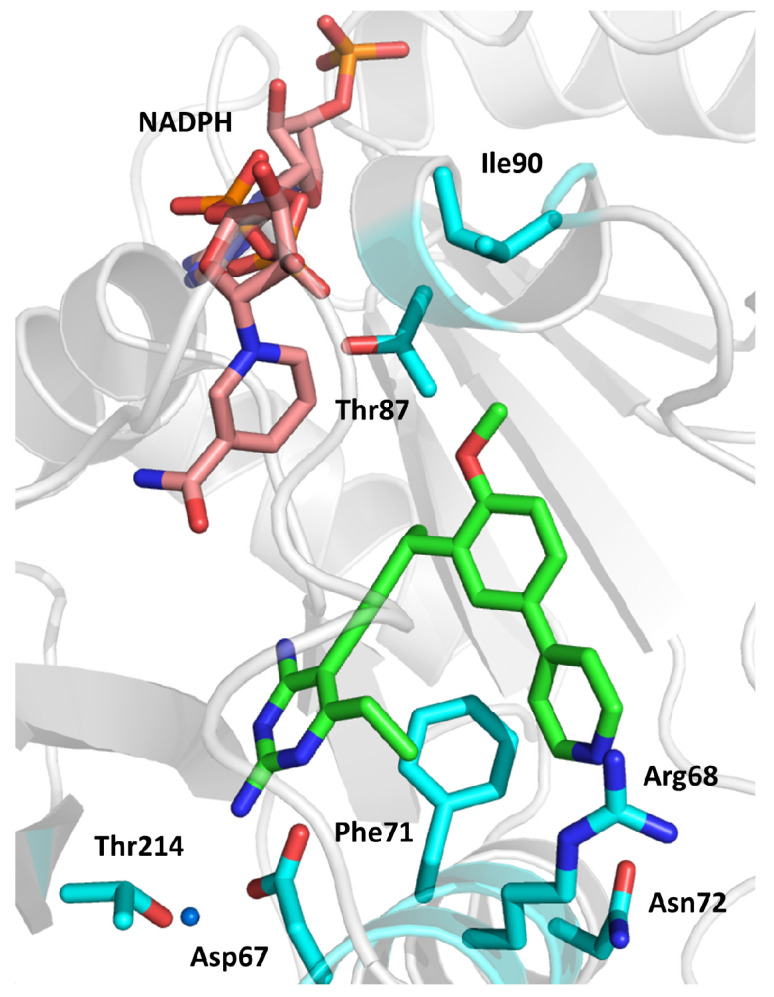
Rv2671 in complex with NADPH (pink sticks) and **30** (green sticks). Interacting residues are represented as cyan sticks, while water molecules are shown as blue spheres (PDB: 6DE5).

Compounds **26** and **30** also displayed a potent inhibition versus Rv2671; Figure 14 shows the binding mode of **30** in the active site of Rv2671, as revealed by the crystallographic studies.

Because the presence of THF is essential to produce dTMP, a crucial building block of DNA, the inhibition of DHFR arrests the growth of mycobacteria by disrupting essential cellular functions, including DNA synthesis and cell wall metabolism.

**Figure 15:**
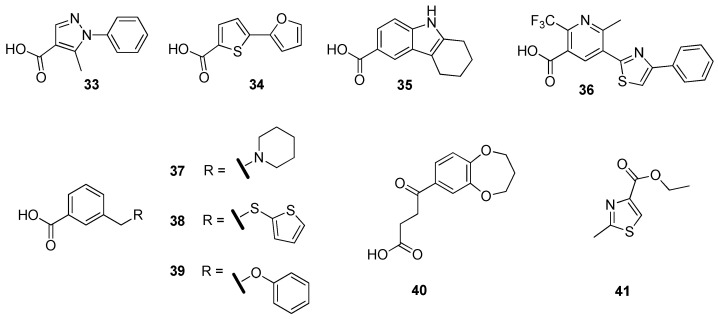
Chemical structure of fragments **33**–**41**.

In 2020, an FBDD approach on DHFR was described [18]. The authors used the complex between a hit and DHFR as the starting point for further elaboration. The screening was carried out on 1250 compounds by DSF; among the 37 positive hits, 30 fragments were screened by 1D STD-NMR, resulting in the selection of 21 fragments, which were characterized by isothermal titration calorimetry (ITC). The compounds showing the best enzymatic affinity are reported in Figure 15. Most of them presented a benzoic acid moiety, which played a crucial role in the interaction with DHFR, probably due to an ionic contact with an arginine residue of the active site.

Fragments **33**–**41** were crystallized in complex with DHFR; the characterization of their interaction with the protein allowed to identify four subregions within the active site groove:

(1) The active site entrance, featuring a positive charge;
(2) The slightly apolar central region of the active site;
(3) The dipyrimidine binding site, characterized by strong negative charges;
(4) The glycerol-binding site, a specific region found in *Mtb* DHFR and not in the human homolog.

**Figure 16:**
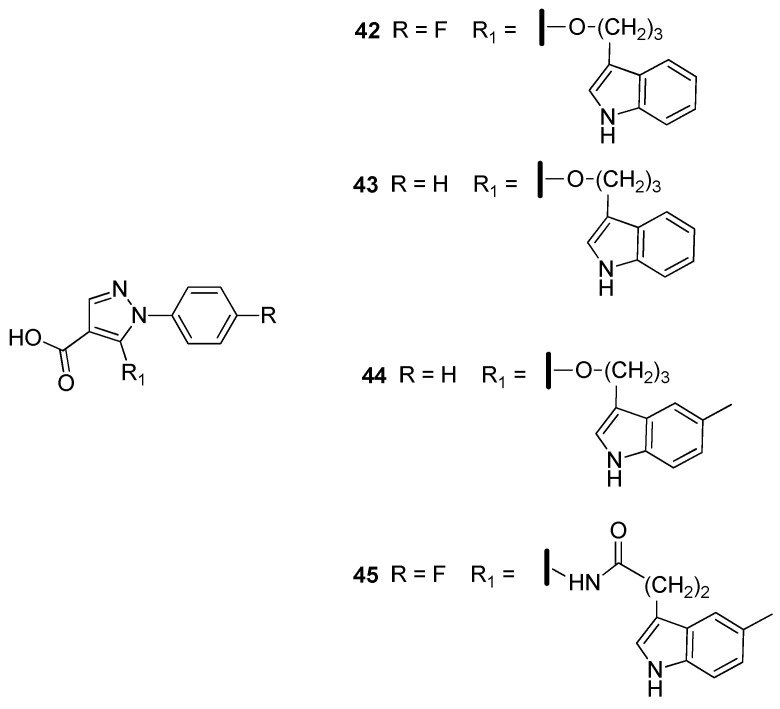
Chemical structure of fragments **42**–**45**.

Because of its selective binding to the glycerol pocket of *Mtb* DHFR, **33** was selected for a SAR investigation, affording the compounds reported in Figure 16. Unexpectedly, the structural analysis of the complexes between DHFR:NADPH and compounds **42**–**45** revealed the absence of interactions with the glycerol-binding pocket. However, these derivatives showed improved affinities for the enzyme (from 600 μM to 17 μM), confirming their potential value as DHFR inhibitors.

### 2.6. Alanine Racemase (Alr)

**Scheme 6:**
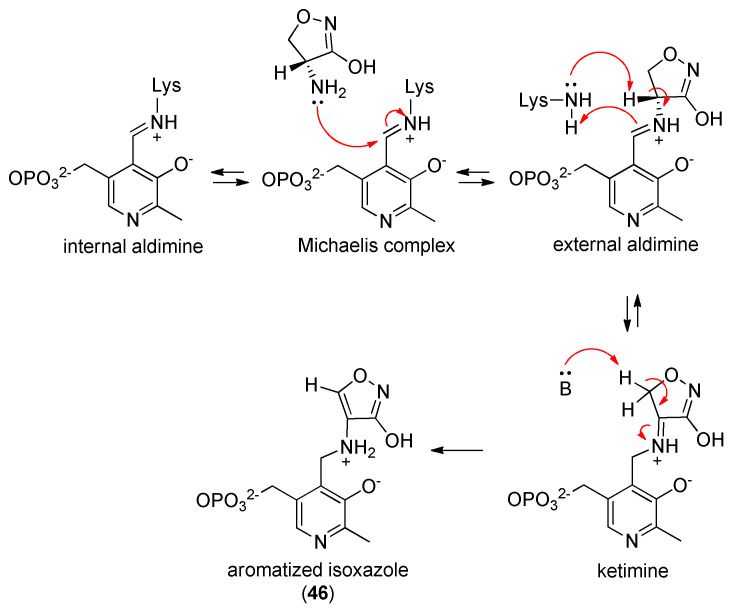
Proposed inhibition mechanism of Alr by DCS.

Alanine racemase (Alr) and D-Ala:D-Ala-ligase (Ddl) are two enzymes involved in the peptidoglycan biosynthetic pathway. d-cycloserine (DCS) is a broad-spectrum antibiotic, used to treat multi- and extensively drug-resistant TB (MRD and XRD-TB), that acts by blocking these two enzymes. The hypothesis that DCS inactivation of Alr is due to the irreversible formation of an adduct with the pyridoxal-5′-phosphate (PLP) is inconsistent with the observation that Alr is active after exposure to chemically relevant DCS concentrations. In an article published in 2020 [19], the mechanism of Alr inhibition by DCS was investigated and explained through the hydrolysis of a DCS-adduct. The authors described the inhibition mechanism (Scheme 6) by the aid of various techniques, including X-ray crystallography.

**Scheme 7:**
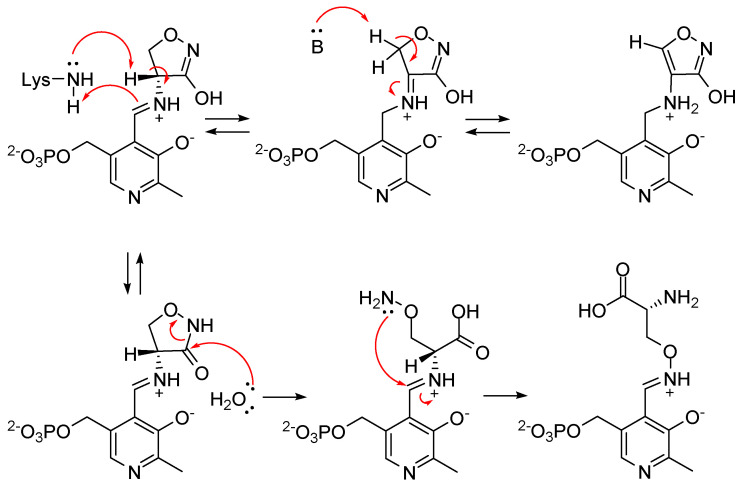
Revised mechanism of DCS-mediated inactivation of Alr.

The subsequent fluorescence analysis revealed the formation of two inactivation products, identified by chemical synthesis and NMR spectroscopy as the isoxazole and the oxime. The isoxazole was in equilibrium with the aldimine, which led to the formation of the oxime, as reported in Scheme 7.

**Figure 17:**
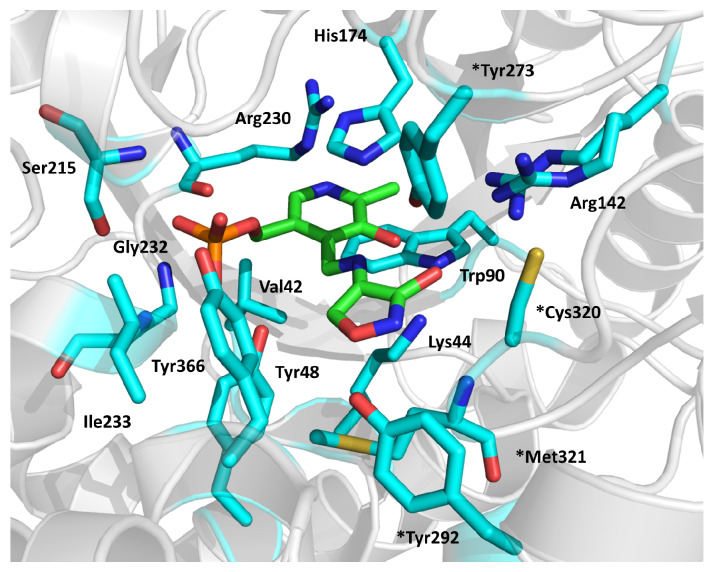
Representation of Alr in complex with **46** (green sticks). Neighboring residues are represented as cyan sticks, while interacting water molecules are omitted for the sake of clarity. The asterisk indicates residues belonging to a nearby chain (PDB: 6SCZ).

The isoxazole-forming pathway, reversible at all steps and accompanied by the formation of the stable oxime, explained the inhibition of Alr and the presence of the two inactivation products. The crystal structure of Alr complexed with **46** (Figure 17) supported the conclusions of this mechanistic study by showing both the external aldimine and the isoxazole in one of the sites and confirming the existence of an equilibrium between the species.

While Alr enzymes from *E. coli* and other fast-growing bacteria are effectively inactivated by DCS, those from *Mtb* (and likely other slow-growing bacteria) are not because of the reversibility of the inhibition mechanism, which likely contributes to Alr reactivation.

### 2.7. l-Aspartate-α-Decarboxylase (PanD)

**Scheme 8:**
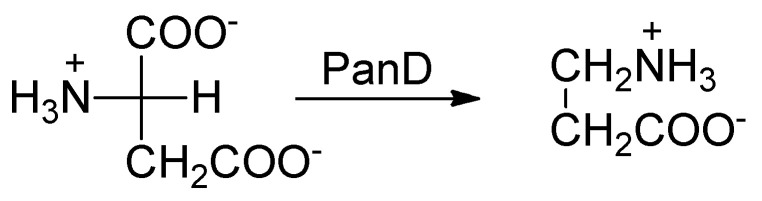
PanD-catalyzed decarboxylation of l-aspartate to β-alanine.

Although pyrazinamide (PZA) has been used for more than fifty years as a front-line anti-TB drug, its mechanism of action is still not fully understood. It is a prodrug converted into the active pyrazinoic acid **47** (POA) by the enzyme pyrazinamidase. Interestingly, several mutations conferring resistance to PZA were located in the l-aspartate-α-decarboxylase (PanD) gene, suggesting that PanD is the target of PZA. PanD catalyzes the decarboxylation of l-aspartate to β-alanine (Scheme 8), as part of the pantothenate biosynthetic pathway, essential for vitamin B_5_ and CoA biosynthesis in *Mtb*.

**Figure 18:**
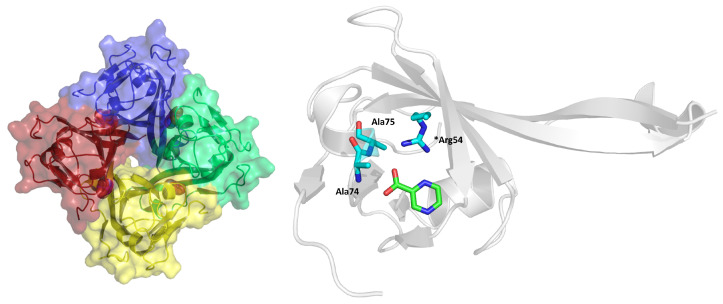
On the **left**, the PanD tetramer, with the four subunits evidenced in different colors (PDB: 6OYY). The ligand **47** is represented as magenta spheres. On the **right**, the interaction of **47** (green sticks) with residues (cyan sticks) in the binding site of PanD; Arg54 belongs to a nearby subunit.

In 2020, Sun and co-workers defined the inhibition of PanD by **47** as competitive, and studied the binding of **47** to PanD by means of X-ray diffraction [20]. The crystal structure of the PanD-**47** complex was solved and refined at 2.7 Å resolution. PanD is a proenzyme that is converted into its active form after a cleavage between Glu24 and Ser25, and the modification of Ser25 to a pyruvoyl group; the active protein consists of a β-chain and an α-chain. In the complex, one molecule of **47** was bound to each active site, located at the interface between two adjacent subunits of the PanD tetramer (Figure 18). The binding pocket of **47** was relatively small (206 Å^3^) and not easily accessible; there was only a narrow tunnel leading from the active site to the inside barrel of the tetramer, with a diameter of about 3.4 Å. Neither aspartate nor **47** fit through the tunnel; therefore, the protein must undergo a conformational change to allow the access of the substrate or the inhibitor to the active site. This conformational modification was consistent with the slow on- and off- rate of the binding of **47**. Four hydrogen bonds contributed to the binding of **47** to PanD; because these contacts are the same as those established by aspartate, **47** was confirmed to be a competitive inhibitor.

In order to investigate the mechanism of resistance to **47**, the authors studied the structures of a resistant mutant, very similar to the wild-type enzyme. None of the mutations involved amino acids that directly contacted **47**; most were on the C-terminal loops of the α and β chains, which seemed to form a flexible lid over the active site.

The reported data allowed to conclude that PanD is the primary target of **47** and that the resistance to PZA derived from alterations in two loops, covering the active site and influencing the affinity, the residence time, and the binding interactions of POA.

### 2.8. Decaprenylphosphoryl-β-d-ribose-2′-oxidase (DprE1)

Arabinose polymers form a large fraction and an essential part of the mycobacterial cell wall. The conversion of decaprenylphosphoryl-β-d-ribose (DPR) to decaprenylphosphoryl-β-D-arabinose (DPA) appears to be the sole source of d-arabinofuranosyl residues in *Mtb*. This transformation is catalyzed by two enzymes: the decaprenylphosphoryl-β-d-ribose-2′-oxidase (DprE1), mediating the FAD-assisted oxidation of the 2′-hydroxyl group of DPR, and the decaprenylphosphoryl-β-d-ribose-2′-reductase (DprE2), which assists the reduction of the 2′-keto group via NAD^+^, affording the hydroxyl epimer DPA.

**Figure 19:**
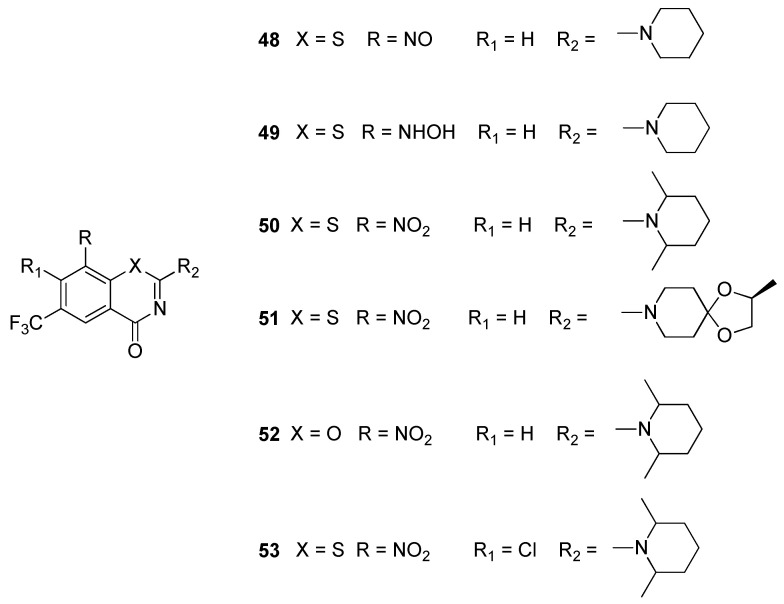
Chemical structure of some DprE1 inhibitors (**48**–**53**).

In 2009, benzothiazinone (BTZ) derivatives were first identified as DprE1 inhibitors [21]. More recently, Richter and co-workers reconsidered these structures publishing an in-depth analysis of the inhibition mechanism of known and new NO_2_-BTZ analogues by means of enzymatic assays, MS spectroscopy, and crystallographic studies [22]. When NO_2_-BTZs enter the catalytic pocket of DprE1, which contains FADH_2_ generated by the oxidation of the substrate, the reduction of nitro to nitroso group results in the formation of a covalent bond with a close cysteine residue. To deepen the study of their mechanism of action and kinetic interaction, several BTZ derivatives were synthesized; using the same approach, new BOZ analogues, in which the sulfur atom in their skeleton was replaced by an oxygen, were prepared and tested (Figure 19).

**Scheme 9:**
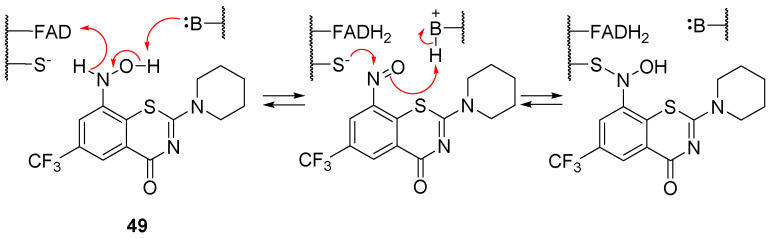
Potential covalent mechanism of DprE1 inhibition by **49**.

The mechanism of the inhibitory activity was studied by means of LC-MS experiments on compound **49**; the inhibitor was incubated with DprE1 in the presence of farnesylphosphoryl-β-d-ribofuranose (FPR), a close analogue of DPR and FAD. The LC-MS analysis of the products confirmed the proposed mechanism, depicted in Scheme 9.

**Figure 20:**
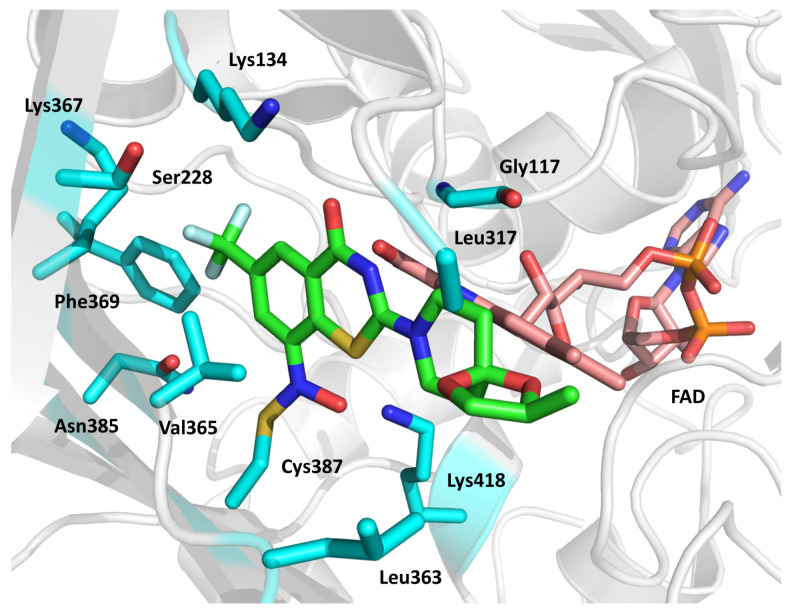
Cocrystal structure of DprE1 with **50** (PDB: 6HEZ). The ligand and the interacting residues are represented as green and cyan sticks, respectively, whereas the FAD cofactor as pink sticks.

The cocrystal structures of DprE1 with the BTZ derivatives **48**, **49**, **50**, and **51**, and with the BOZ compound **52** were all solved with two DprE1-inhibitor complexes in the asymmetric unit (Figure 20). The five compounds formed a covalent bond with the cysteine present in the active site (Cys387), while the trifluoromethyl group faced the same residues; only subtle variations in the orientation of the compounds were reported, as well as non-covalent contacts with DprE1. Notably, despite the similar enzyme-ligand contacts, the inhibitory activity underwent considerable variations. The authors concluded that the mechanism of action detailed in their study may support the development of a next generation of DprE1 inhibitors.

### 2.9. Cytochromes CYP124 and CYP121

*Mtb* has evolved a sterol catabolizing system, which is believed to be essential for its virulence; hence, the enzymes involved in the catabolism of host cholesterol are validated targets for anti-TB drug discovery. The cholesterol catabolism is initiated by the 3β-hydroxysteroid dehydrogenase (3βHSD) and by three monooxygenases (CYP142, CYP125, and CYP124). Cholesterol is not an essential source of nutrition for *Mtb* during infection, suggesting that these enzymes have a physiological role beyond cholesterol metabolism. Cholesterol derivatives, like oxysterol and vitamin D_3_, are important factors of the human immune system, able to directly regulate the inflammatory program of macrophages and to participate in the establishment of the immune response.

**Figure 21:**
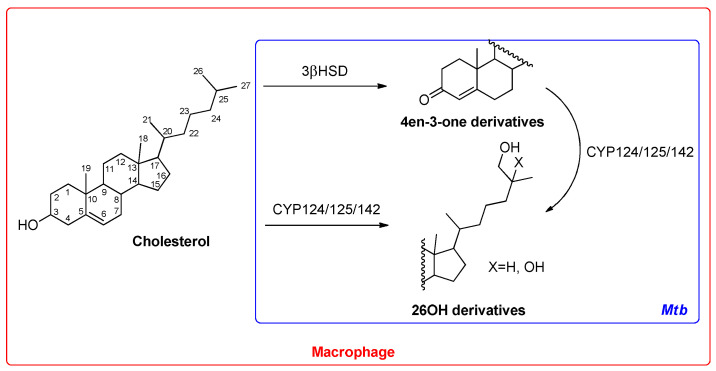
Simplified depiction of the metabolism of host sterols by *Mtb* enzymes.

In 2021, Varaksa and co-workers studied cholesterol metabolism in *Mtb* not only as a source of carbon and energy, but also as a possible way to degrade immunoactive cholesterol derivatives [23]. The authors reported that 3βHSD and CYP124, CYP125, and CYP142 can metabolize human immunoactive oxysterols in vitro, and identified the corresponding metabolic products, deriving from the oxidation of the hydroxyl moiety at the 3-position and the introduction of a hydroxyl group at the 26-position (Figure 21). Through spectrophotometric titration and activity assays, a series of sterol substrates of these enzymes were identified; the attention was focused on 25-hydroxy-cholesterol, a potent bioactive lipid in both adaptive and innate immune system.

**Figure 22:**
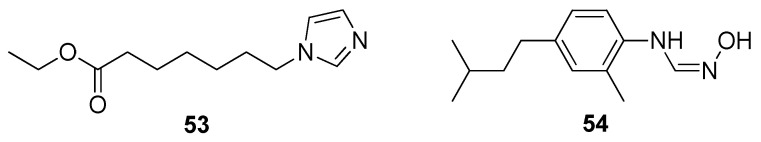
Chemical structure of **53** and **54**.

Among the three monooxygenases, CYP124 is the most active and, for this reason, its complex with cholestenone was studied by X-ray diffraction at 1.65 Å resolution. A binding tunnel with 24 lining residues was defined [21]; this passage narrows toward the catalytic site to tightly enclose the aliphatic side chain, and it opens in the opposite direction to create more space for the tetracycle. Since CYP124 efficiently converts 1α-hydroxy-derivatives of ergocalciferol (vitamin D_2_) and cholecalciferol (vitamin D_3_) to their hydroxy products, the structures of CYP124 in complex with secosteroids were solved and carefully described in the same article. To test the effect of the suppression of the enzymes involved in the degradation of oxysterols, two imidazole derivatives (CHImi, **53**, and cpd5′, **54**, Figure 22), known inhibitors of many CYPs, were studied on CYP124.

**Figure 23:**
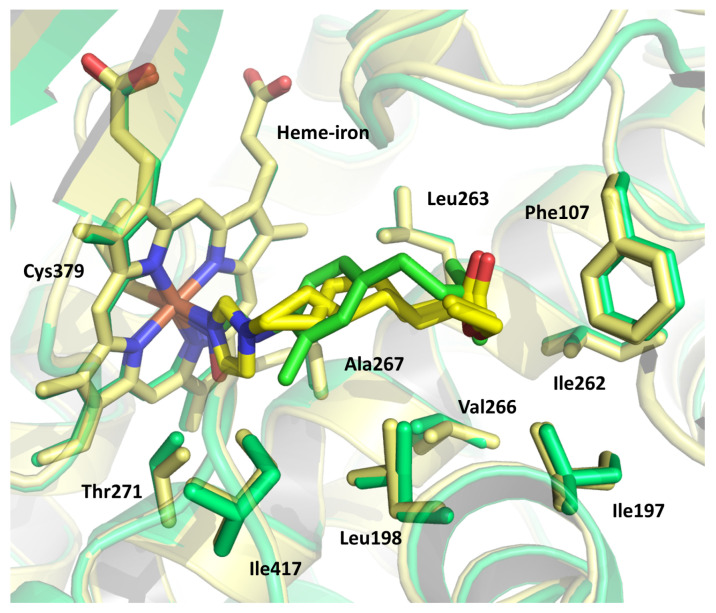
Superimposition of the active sites of CYP124 complexed with **53** (yellow, PDB: 6T0K) and **54** (green, PDB: 6T0L). The ligand and interacting residues are represented in sticks.

The structures of **53** and **54** in complex with CYP124 were solved and discussed in the article (Figure 23) [24].

The observation that steroid-metabolizing enzymes can hydroxylate several human immunoactive molecules, starting from cholesterol, may pave the way to new pharmacological approaches aimed at breaking dangerous interrelations between *Mtb* and the host immune system.

**Scheme 10:**
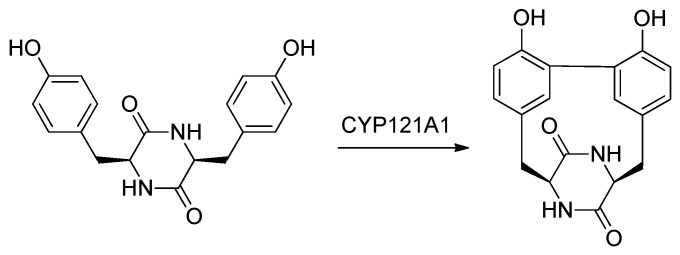
CYP121-catalyzed biosynthesis of mycocyclosin.

Among other mycobacterial cytochromes, CYP121 has sparked the interest of the medicinal chemistry community because it is a very peculiar enzyme, absent in other microorganisms or mammals. CYP121 catalyzes the oxidative crosslinking of the two tyrosines of a Tyr-Tyr cyclodipeptide, affording mycocyclosin (Scheme 10).

**Figure 24:**
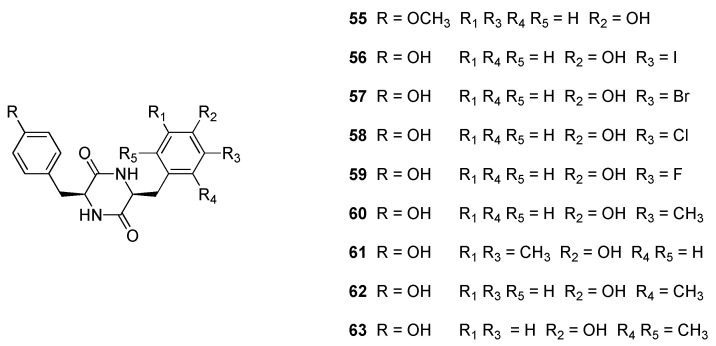
Chemical structure of compounds **55**–**63**.

The gene encoding CYP121 is essential, but the role of cyclotyrosine in *Mtb* biology has yet to be determined. The unusual mycocyclosin and the oxidative reaction catalyzed by CYP121, affording the formation of a C-C bond instead of the usual introduction of an oxygen atom, prompted the study of analogues of the natural substrate, as possible inhibitors of this enzyme. Previously, an anilinophenylpyrazole derivative had displayed a nanomolar affinity for CYP121 (*K_d_* = 15 nM). Starting from this result, Rajput and collaborators synthesized a set of derivatives, investigating the role of the tyrosyl phenolic groups in the binding affinity and modifying the 4-position of the aromatic ring [24]. Analogues lacking one or both phenolic groups or having one or both transformed into the corresponding methyl ether, were prepared. The phenolic groups were replaced with halogens; halogens or methyl groups were introduced at the *ortho* or *meta* positions (Figure 24).

**Figure 25:**
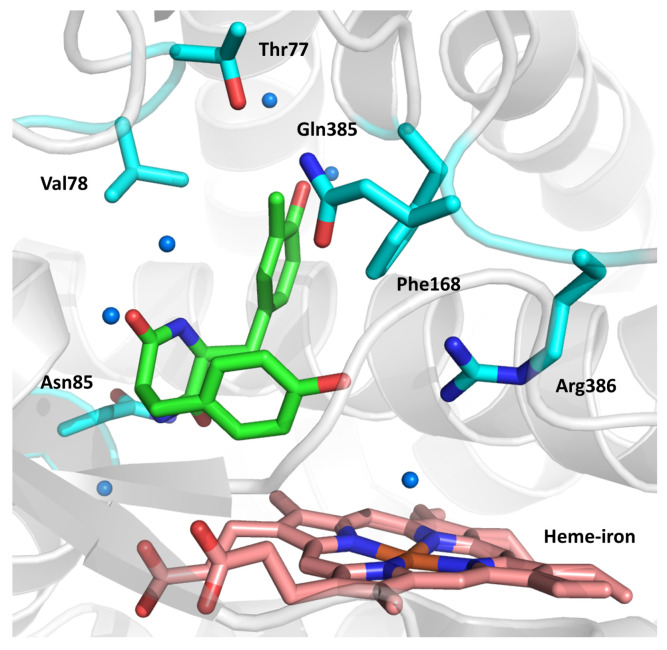
X-ray crystal structure of **60** (green sticks) bound to CYP121 (PDB: 6RQ0). Interacting residues are shown as cyan sticks, while water molecules are represented as blue spheres. The heme group is depicted as pink sticks.

The interaction of the natural substrate and its analogues with CYP121 were analyzed by UV-VIS spectroscopy and electron paramagnetic resonance spectroscopy. X-ray crystal structures of **55**–**63** bound to CYP121 were also obtained and described [24]. The binding of the 3-methyl analogue of **55**, compound **60**, within the active site of the enzyme, is illustrated in Figure 25. The compound was accommodated in a binding pocket distal to the heme group, without involving steric clashes or inducing conformational changes, forming hydrophobic interactions with Phe168 and Val78. The carbonyl group of the diketopiperazine ring formed a direct hydrogen bond with the side chain of Asn85. Moreover, the binding of the ligand did not displace the water molecule associated with the Fe(III) ion; in addition, more water molecules contacted the phenolic groups of **60** and Thr177, Glu385, and Arg386. The detailed descriptions of the complexes between CYP121 and other analogues were also reported [24].

Overall, the interaction pattern was highly conserved. Notably, in the complex with the 3-iodoanalogue **56**, the iodine atom was very close to the Fe(III) ion of the heme group, filling the space otherwise occupied by a water molecule. The phenolic group of the substituted aromatic ring was within the hydrogen-bonding distance of Arg386, indicating a tight binding that well correlates with the high affinity observed for this compound. The specific interaction between the iodine atom and either the Fe(III) ion or the heme group may also contribute to the high binding affinity detected for this compound.

Combining the knowledge about the structural data and the biological roles the two CYPs here described can be considered a promising strategy for the development of novel therapeutic candidates.

A non-CYP enzyme involved in cholesterol catabolism is HsaD, an α,β-hydrolase belonging to the *meta*-cleavage product (MCP) subfamily. It catalyzes the hydrolytic cleavage of a carbon-carbon bond in 4,5–9,10-diseco-3-hydroxy-5,9,17-tri-oxoandrosta-1(10),2-diene-4-oic acid (DSHA), yielding 9,17-dioxo-1,2,3,4,10,19-hexanorandrostan-5-oic acid (DOHNAA) and 2-hydroxy-hexa-2,4-dienoic acid (HHD). HsaD has been shown to be essential for the survival of *Mtb* within macrophages and overexpressed in hypervirulent strains of *M. bovis* BCG. This interesting target was explored in an FBDD campaign in 2017. Some interesting fragments were identified, modified to define the SAR, and cocrystallized with the enzyme to determine the binding mode and pave the way for the design of more complex molecules. Some of the fragments also exhibited a promising antitubercular activity and a negligible toxicity on mammalian cells, confirming their potentiality as starting points for the design of new antitubercular agents [25].

### 2.10. Ser/Thr Protein Kinase B (PknB)

Targeting essential virulence factors, like protein kinases involved in signal transduction, is considered a very promising approach. In bacteria, an important transmembrane signaling system is regulated by the receptor-type serine/threonine protein kinases (STPKs). PknB belongs to the transmembrane cluster of STPKs: its catalytic domain is at the N-terminus, linked to the C-terminal sensor domain through a single transmembrane helix. PknB plays a fundamental role in cell shape regulation and hypoxia-induced cellular replication, and it is essential for the growth and survival of *Mtb* in the host; hence, it can be regarded as a very attractive drug target. The activation mechanism involves the phosphorylation of Ser/Thr residues in the activation loop upon ATP binding to the active site, leading to a conformational change from the catalytically inactive dephosphorylated state to the active phosphorylated form. The dimerization, promoted by the binding of the sensor domain to a ligand, stabilizes the active conformation of the kinase domain, facilitating intermolecular autophosphorylation, thus producing a fully active kinase domain able to phosphorylate the substrates. Finally, the dephosphorylation by PstP restores the inactive unphosphorylated monomeric form [26].

PknB was the first *Mtb* kinase for which the crystal structure of the catalytic domain was described [27]. Most of the published works deal with compounds inhibiting the kinase in the micro and sub-micromolar range, but with limited activity on mycobacteria.

**Figure 26:**
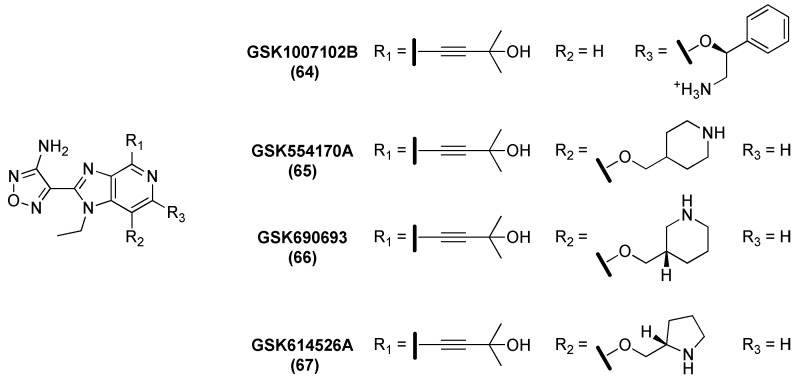
A selection of the most active compounds (**64**–**67**) belonging to the IPA class.

In 2018, Wlodarchak and co-authors identified a new family of imidazopyridine aminofurazans (IPAs) as PknB inhibitors by an in silico screening. A selection of the most interesting IPA candidates is presented in Figure 26 (**64**–**67**). When co-administered with β-lactams, these compounds worked at lower concentrations, differently from the antibiotics traditionally used to treat mycobacterial infections; this is consistent with the role of PknB in sensing cell wall stress. Among them, GSK690693 (**66**) showed sub-micromolar affinity to the kinase (IC_50_ = 0.34 µM) and significantly lowered its antimycobacterial activity on *M. smegmatis* or *M. bovis* BCG (bacillus Calmette-Guerin) in association to sub-MIC_50_ concentrations of meropenem, suggesting a synergistic action between PknB inhibitors and β-lactams. The available crystal structure in complex with the target (PDB: 5U94) displayed conserved features in its binding mode to the PknB hinge region [28].

**Figure 27:**
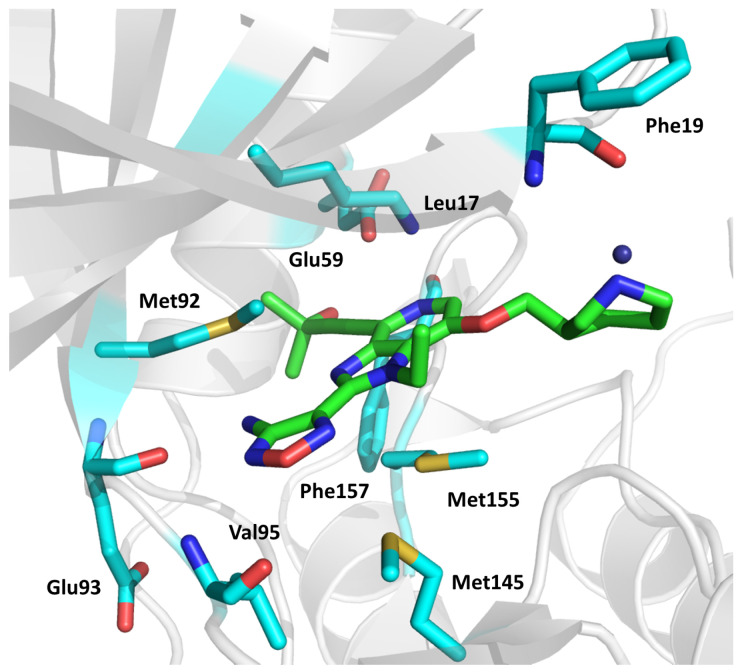
X-ray crystal structures of **66** (green sticks) bound to PknB (PDB: 5U94). Interacting residues are shown as cyan sticks, while water molecules are depicted as blue spheres.

The structure of the PknB-**66** cocrystal confirmed that the inhibitors are bound as predicted (Figure 27). **66** overlapped the ATP binding position, with the aminofurazan ring aligning with the adenine ring of ATP. The aminofurazan ring made two hydrogen bonds to the backbone of the hinge region of PknB (Glu93 and Val95), and the dimethylpropargyl alcohol made two hydrogen bonds to the back pocket via the backbone of Phe157 and the side chain of Glu59. The piperidine ring made a weak hydrogen bond to the backbone of the P-loop (Phe19) through an ordered water, whereas the nitrogen of the pyridine ring formed a hydrogen bond with the catalytic lysine. Stacking interactions involved Met145 and Met155 with the imidazopyridine core, and Met92 with the aminofurazan ring. The ligand binding pose was also consolidated by eight hydrophobic interactions.

These data contributed to deepen the knowledge of mycobacterial signaling pathways in *Mtb*, in which Ser/Thr kinases are actors of a complex interplay. Therefore, these structure-based outcomes maintain a strong potential for the future development of new anti-tubercular drug candidates.

### 2.11. Adenosine Kinase (AdoK)

Adenosine kinase (AdoK) is an attractive target for the development of new antimycobacterial agents because it belongs to the purine salvage pathway, whose efficient functioning is essential for *Mtb* survival. Moreover, this enzyme has an important role in the latent state, since *Mtb* must recycle bases and/or nucleosides to survive in the hostile environment imposed by the host. Notably, the human homolog (hAdoK) has a low sequence identity (<20%) to *Mtb* AdoK and exhibits several unique physicochemical properties. Taken together, these peculiarities support the choice of AdoK as a target for the search of new antibacterial drugs.

**Scheme 11:**
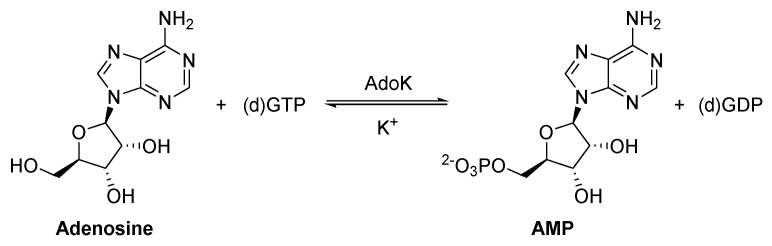
Phosphorylation of adenosine to AMP, catalyzed by adenosine kinase (AdoK).

AdoK catalyzes the conversion of adenosine to AMP; the enzyme activity is dependent on the presence of the Mg^2+^ ion and ATP (Scheme 11).

High-resolution structures for AdoK (PDB: 2PKM, 2PKF, 2PKK, and 2PKN) demonstrated that the apo form of the enzyme has the active site in an open conformation; after adenosine binding, a lid domain undergoes a large conformational change, which leads to the formation of interactions with the substrate and residues of the active site.

The purine salvage pathway in *Mtb* still leaves unexplored possibilities for drug development; therefore, structural data of enzyme-inhibitor complexes may provide a broader knowledge to drive the future search for chemical agents with novel mechanisms of action and a more selective biological activity.

**Figure 28:**
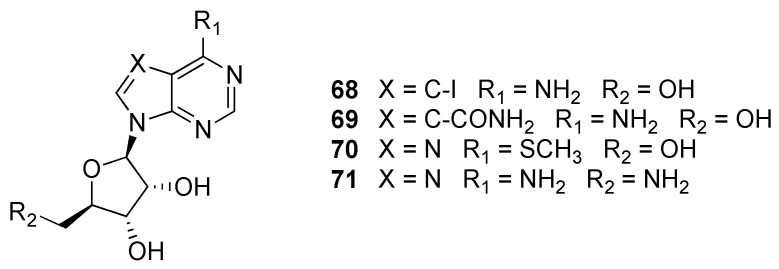
Chemical structure of compounds **68**–**71**.

Recently, Crespo et al. disclosed very potent and safe AdoK inhibitors by means of a structure-based drug design (SBDD) approach [29]. Several 6-substituted adenosine analogues complexed with AdoK, displaying high anti-*Mtb* activity in a whole-cell assay, were crystallized and solved revealing key insights into the region surrounding the active site. The crystal structures of the target with iodotubercidin (**68**, PDB: 6C67) and sangivamycin (**69**, PDB: 6C9N) gave information about the conformational flexibility and chemical properties of the area surrounding the binding pocket, near the N7-position of the adenosine scaffold. The complexes with 6-methylmercaptopurine riboside (**70**, PDB: 6C9P) and 5′-aminoadenosine (**71**, PDB: 6C9Q) allowed to explore the 6- and 5′-positions, respectively, providing a structural explanation behind the specificity of the N6-substituted adenosine analogues (Figure 28).

**Figure 29:**
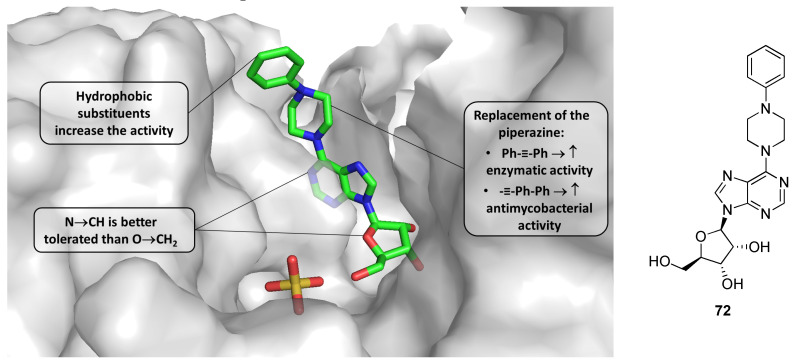
Active site of AdoK complexed with **72** as green sticks (PDB: 6C9V). The boxes summarize the SAR obtained for this class of compounds.

Improved derivatives were prepared by Crespo et al. based on the structural data, which suggested the presence of a size threshold conferring specificity to the active site vs the ATP site. This observation could be useful for the future design of bisubstrate-like inhibitors characterized by small substituents at the N6-position and bulky groups at the 5′-position, which increase the number of favorable contacts with the enzyme and sterically prevent the full closure of the lid domain [29]. Figure 29 reports the crystal structure of a second-generation inhibitor (**72**), together with a summary of the SAR obtained for this set of compounds.

### 2.12. Thymidylate Kinase (TMPK)

In recent years, the thymidylate kinase (TMPK) has been selected as a target for SBDD due to its essential role in DNA biosynthesis and because the configuration of its active site is unique in the TMPK family [30]. Crystallographic analyses supported the characterization of the substrate binding mode and the definition of the catalytic mechanism, which involves the ATP-dependent phosphorylation of deoxythymidine 5‘-monophosphate (dTMP). In particular, the structural data were pivotal to elucidate the process of lid closure upon ligand binding and determine the unusual location of the Mg^2+^ ion, which coordinates TMP in the active site pocket (PDB: 1W2G and 1N5K).

**Figure 30:**
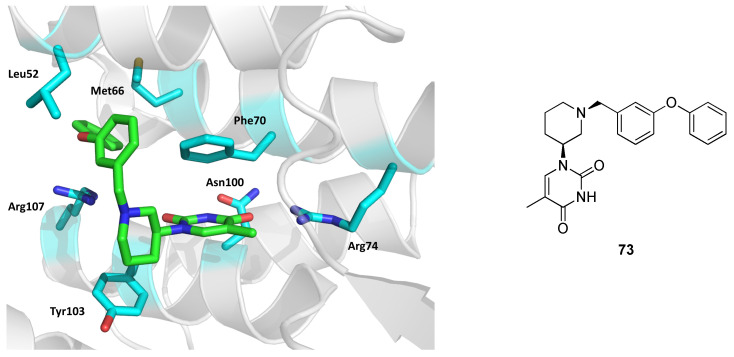
On the **left**, representation of the cocrystal structure of **73** (green sticks) bound to TMPK (PDB: 5NQ5). Interacting residues are shown as cyan sticks. On the **right**, chemical structure of **73**.

Guided by enzyme assays and by the X-ray characterization of the target, Merceron et al. performed rational modifications on a series of ligands, disclosing very potent inhibitors [30]. The researchers were inspired by the cocrystal structure of TMPK in complex with **73** (IC_50_ = 10 μM, Figure 30): the thymine ring of the inhibitor was deeply located into the catalytic pocket, forming a π−π stacking interaction with Phe70 and hydrogen bonds between O4 and N3 and Arg74 and Asn100, respectively; the binding was further stabilized by hydrophobic interactions with the target.

**Figure 31:**
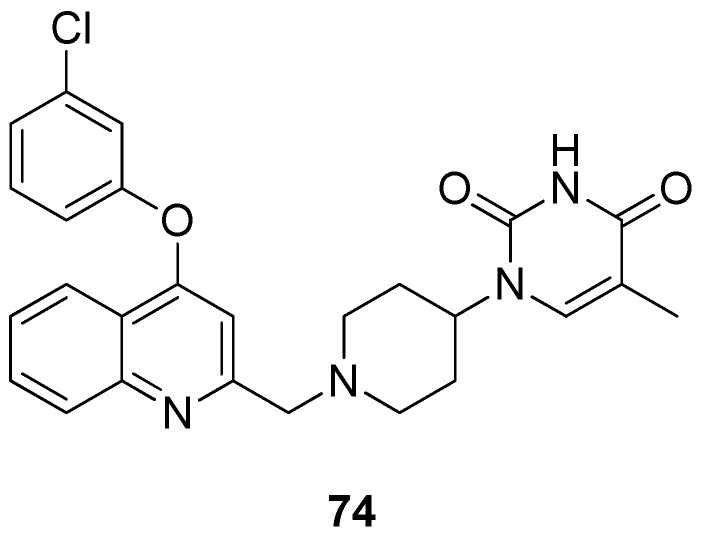
Chemical structure of compound **74**.

The analysis of the ligand efficiency resulted in two novel chemical series: one constituted by phenoxybenzyl analogues, and the other by quinolin-2-yl analogues. Chemical explorations led to the phenoxylquinolin-2-yl derivative **74** (Figure 31), which exhibited a potent TMPK inhibitory activity and a good antimycobacterial activity (IC_50_ = 0.95 μM, MIC_99_ = 12.17 μM against *Mtb* H37Ra).

**Figure 32:**
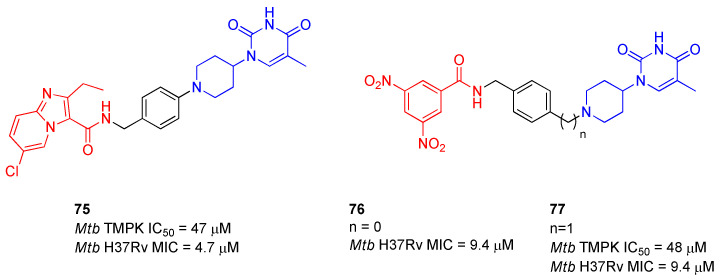
Chemical structure and biological properties of compounds **75**, **76**, and **77**. The three portions of the compounds are evidenced in red, black, and blue.

With the aim of improving the MIC, Jian et al. [31] synthesized new hybrid molecules, introducing an imidazo[1,2-a]pyridine or a 3,5-dinitrobenzamide moiety, which are known to increase bacterial uptake [32]. This strategy afforded the safe analogues **75**, **76**, and **77** (Figure 32), having moderate TMPK inhibitory potencies, and antimycobacterial activities in the low-micromolar range.

**Figure 33:**
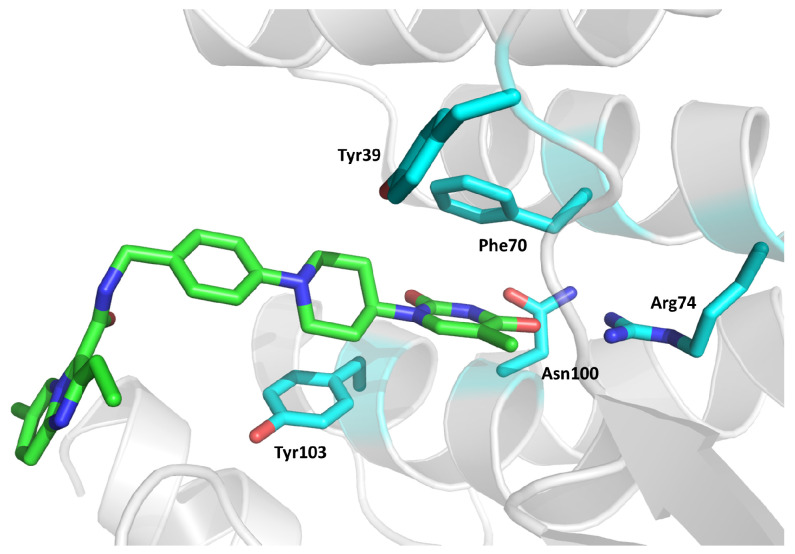
View of the binding mode of compound **75** (green sticks) into the TMPK active site (PDB: 6YT1). Interacting residues (carbon atoms in cyan) are depicted as sticks.

Since **75** was structurally different from the previously studied derivatives, the authors solved its cocrystal structure with TMPK, obtaining data at high resolution (PDB: 6YT1), which showed that **75** adopted an L-shaped conformation, with the amide tail bent toward the α7-helix (Figure 33). The inhibitor formed π−π stacking interactions, connecting its heterocyclic system to Tyr39, Tyr103, and Phe70, and established polar contacts with Arg74 and Asn100. In addition, two antiparallel-oriented molecules of **75** contacted residues of a symmetry-related protomer, involving Tyr39 and His53. The amide tail did not show specific interactions, possibly accounting for the moderate inhibitory activity of the compound. As for the 3,5-dinitrobenzamide derivatives, **76** displayed solubility issues in the TMPK enzymatic assay, whereas **77** exhibited a similar inhibitory potency to **75**.

The SBDD approach adopted in this study proved to be a valid strategy for the discovery of new antimycobacterial agents.

### 2.13. tRNA (guanine37-N^1^)-methyltransferase (TrmD)

**Scheme 12:**
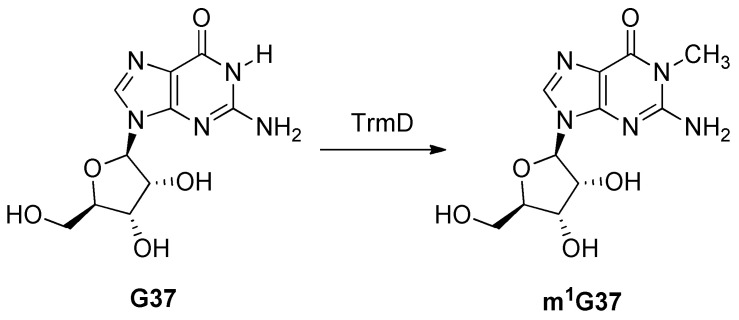
Synthesis of m^1^G37-tRNA, catalyzed by TrmD.

The tRNA (guanine37-*N*^1^)-methyltransferase (TrmD) is an essential enzyme in many bacterial pathogens. It catalyzes a methyl transfer from *S*-adenosyl-l-methionine (SAM) to the guanine N^1^ at nucleotide position 37 in a subset of bacterial t-RNA isoacceptors (Scheme 12) [33]. The modified guanosine is essential for the maintenance of the correct reading frame during translation [34].

Trm5, the functional homolog of TrmD in eukaryotes, has a dissimilar active site and binds SAM differently compared to TrmD, suggesting that TrmD could be an attractive target to develop antimicrobial agents.

**Figure 34:**
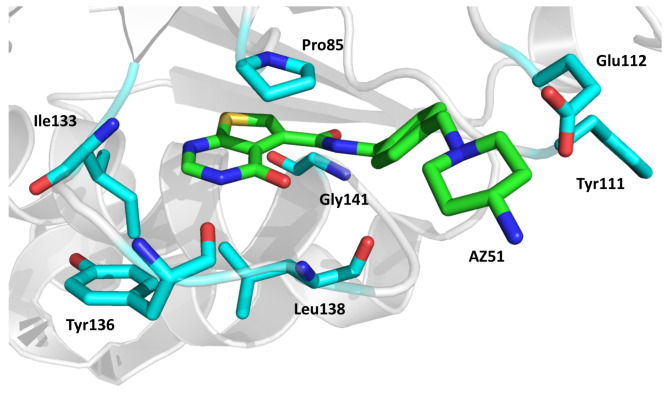
Main interactions established in the active site of TrmD by **78**, in green sticks (PDB: 6JOF). Interacting residues are shown as cyan sticks.

Despite AZ51 (**78**), a thienopyrimidinone studied by Hill et al. in 2013, binds with low affinity to the TrmD enzyme of mycobacteria and Gram-positive bacteria, the crystal structure of *Mtb* TrmD with **78** was successfully obtained at a resolution of 2.2 Å [33]. This compound bound to the *S*-adenosyl-l-homocysteine (SAH) binding site without inducing conformational changes and interacted with three active-site loops of the enzyme (Figure 34).

**Figure 35:**
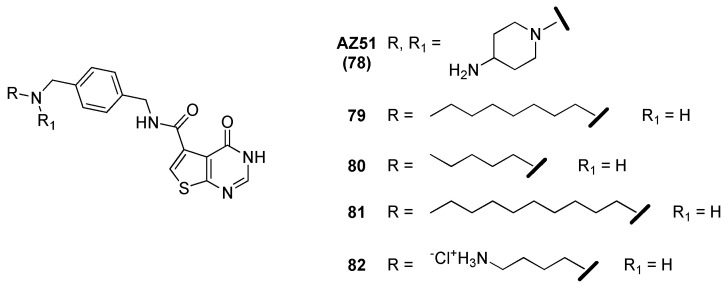
A selection of the most active thienopyrimidinone-based TrmD inhibitors (**78**–**82**).

The cocrystal structure of **78** with *Mtb* TrmD was compared to that obtained with the homolog from *Pseudomonas aeruginosa* (*Pa* TrmD). The residues involved in the interaction with **78** were strictly conserved. However, the binding of the compound did not induce the flip of the side chain of Tyr111 in *Mtb* TrmD, while the corresponding residue (Tyr120) in *Pa* TrmD turned 180° to form stacking interactions with the phenyl ring of the inhibitor, suggesting a more rigid active site in *Mtb* TrmD compared to *Pa*TrmD. In the absence of the conformational changes at the wall loop, the piperidine ring of **78** was positioned differently in *Mtb* TrmD, resulting in the loss of interactions between the terminal amine and the side chain of Glu112. These structural studies prompted the synthesis of several thienopyrimidinone derivatives, showing nanomolar activity against TrmD; the best candidates (**79**–**82**) are reported in Figure 35 [33].

**Figure 36:**
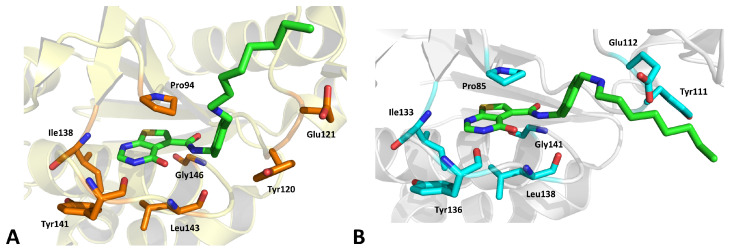
Crystal structures of **79**, in green sticks, in complex with *Pa* TrmD (**A**, PDB: 5ZHN) and *Mtb* TrmD (**B**, PDB: 5ZHL). Interacting residues are visualized as colored sticks.

The cocrystal structures of these compounds showed that they adopted similar binding modes in both *Mtb* TrmD and *Pa* TrmD, by interacting with conserved residues. The most significant discrepancy lay in their different ability to induce a conformational change in the active-site wall loop and the flip of the Tyr side chain, which plays an important role in inhibitor binding. Figure 36 shows the active-site residues of *Pa* TrmD and *Mtb* TrmD involved in the interactions with the potent inhibitor **79** [33].

### 2.14. Nicotinic Acid Mononucleotide (NaMN) Adenylyltransferase (NadD)

**Scheme 13:**
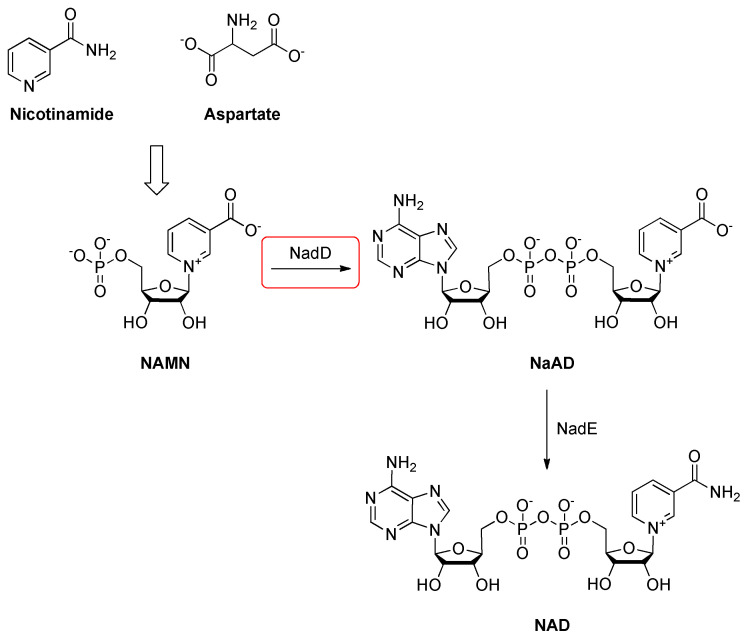
Biosynthetic pathway leading to the production of NAD in mycobacteria.

Bedaquiline (BDQ), one of the few drugs recently approved for the treatment of TB, acts by targeting ATP synthase, thus blocking the production of ATP. However, this essential nucleoside can be obtained by other pathways, including glycolysis. Despite being a less efficient process, glycolysis produces ATP faster than oxidative phosphorylation. Unsurprisingly, the use of BDQ has been associated with an upregulation of glycolytic enzymes. Notably, the NAD cofactor plays an essential role both as an electron donor in the respiratory chain and as a key oxidant in glycolysis. Hence, its depletion effectively hinders the two main sources of ATP in the mycobacterial cell. Among the key enzymes involved in the biosynthesis of NAD (Scheme 13), NaMN adenylyltransferase (NadD) has been recently validated as a new drug target for latent and active TB, due to its considerable divergence to the human counterpart (NMNAT1−3) [35].

**Figure 37:**
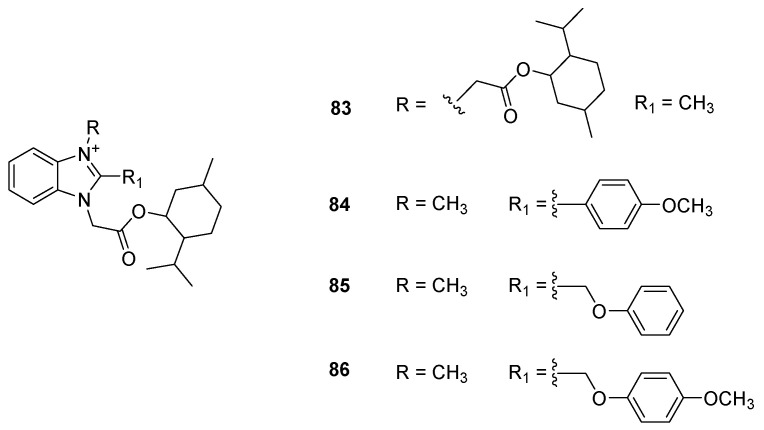
Chemical structure of compounds **83**–**86**.

**Figure 38:**
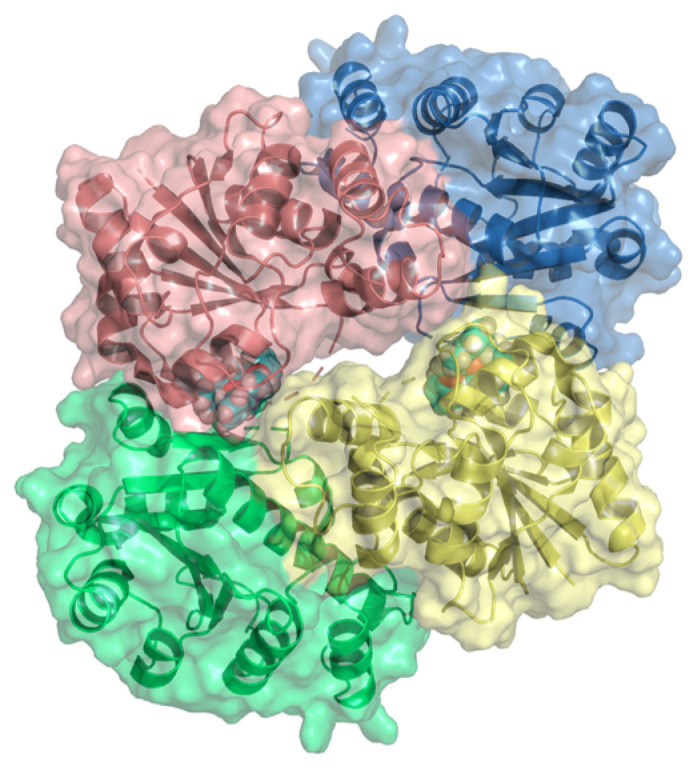
Structure of NadD in complex with **85** (PDB: 6BUV). Chains A and A’ are depicted in green and blue, while chains B and B’ are in yellow and red. A space-filling model is used to represent the inhibitor molecules (cyan).

In an attempt to identify new inhibitors of NadD, ~1400 bioactive compounds were screened against the recombinant NadD, leading to the identification of a new class of benzimidazolium derivatives endowed with bactericidal activity on different mycobacteria, including *M. abscessus*, MDR-*Mtb*, and dormant *M. smegmatis* [35]. Starting from the originator (**83**), a library of derivatives was designed and synthesized; the best compounds (**84**–**86**) are reported in Figure 37. The cocrystal structure of NadD with compound **85** (Figure 38), determined at 1.86 Å resolution, revealed that the binding of the inhibitor induced the formation of a new quaternary structure in which two copies of the inhibitor occupied symmetrical positions at the dimer interfaces [35].

The structure of the inhibitor–target complex evidenced that the binding sites were formed by chains B and A’, and chains B’ and A, respectively, and **85** occupied a hydrophobic pocket, exhibiting high steric complementarity with the surface of the two enzyme chains.

The menthol moiety of the inhibitor made van der Waals interactions with several hydrophobic residues of the pocket, including Leu151 and Leu158 from chain A’, while Glu160 contributed to the positioning of the positively charged benzimidazole core. The phenoxymethyl moiety was oriented away from the protein, allowing to accommodate various potential substituents at the R_1_ position [35].

Notably, the class of compounds derived from **85** showed a strong bactericidal effect against *Mtb*, including MDR-, XDR-, and TDR-strains, and a significant bactericidal effect under nonreplicating conditions in *M. smegmatis*. Therefore, the inhibition of NadD represents a viable strategy to kill dormant and MDR strains, also in combination therapies.

### 2.15. Enhanced Intracellular Survival (Eis) Transferase

Kanamycin (KAN) and amikacin (AMK) belong to the class of the aminoglycoside antibiotics, injectable second-line agents commonly used for the treatment of resistant TB infections. In recent years, resistance to both KAN and AMK has emerged in some *Mtb* isolates, gradually becoming a marker of XDR-TB, along with an insensitivity to fluoroquinolones. The principal mechanism of inactivation of these drugs is based on the overexpression of the Eis protein, an acetyltransferase that catalyzes the multi-acetylation of aminoglycosides at different positions. While a single modification is not sufficient to abolish the antibiotic effect, the wide and complex Eis active site can determine an efficient acetylation on multiple sites and on a large variety of substrates. For this reason, the use of novel Eis inhibitors in association with KAN or AMK could be a valid approach to counteract the spreading resistance to aminoglycosides.

**Figure 39:**
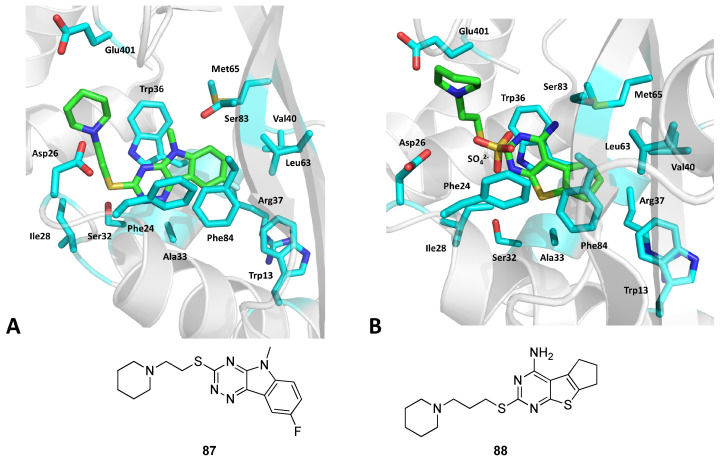
Zoomed-in views of the binding sites of the complexes between Eis and **87** (**A**, PDB: 6B3T), and **88** (**B**, PDB: 6VUZ). The ligands and interacting residues are represented as green and cyan sticks, respectively.

An HTS screening on ~23,000 molecules led to the identification of a new class of Eis inhibitors containing a thieno[2,3-d]pyrimidine moiety. As a reference compound, Punetha et al. [36] chose a molecule containing a 1,2,4-triazino[5,6b]indole-3-thioether core (PDB: 6B3T), partially isosteric with the tricyclic moiety of the new class of inhibitors [37]. The crystal structures of Eis in complex with 12 ligands showed that these molecules occupied the aminoglycoside binding pocket, inhibiting the acetyl transfer with a competitive mechanism. The tricyclic moieties of all compounds were sandwiched between Trp36 and Phe84, but differently oriented with respect to the moiety of the reference compound **87** (Figure 39). The most hydrophobic ring, unsubstituted or modified by small nonpolar or weakly polar groups, was in contact with the lipophilic wall of the binding pocket (lined by Trp13, the aliphatic portion of Arg37, Val40, Leu63, and Met65), whereas the large substituent on the opposite ring extended along the substrate binding cleft toward the solvent, a general direction of the thioether side chain.

The crystal structure of **88** (IC_50_ = 0.75 ± 0.06 μM; PDB: 6VUZ, Figure 39) with Eis revealed that the linker placed the positively charged amino group of the piperidine equidistant from the carboxyl groups of Asp26, Glu401, and the C-terminal carboxyl group, forming strong salt bridges. The piperidine ring was found in different conformations, all related by rotations along the C−N bond connecting the ring to the linker.

Some compounds of this series revealed toxicity against *Mtb* and mammalian cells, while **88** was nontoxic to *Mtb*, but potent in Eis inhibition and endowed with synergic activity with KAN, emerging as a promising compound for further studies [36].

**Figure 40:**
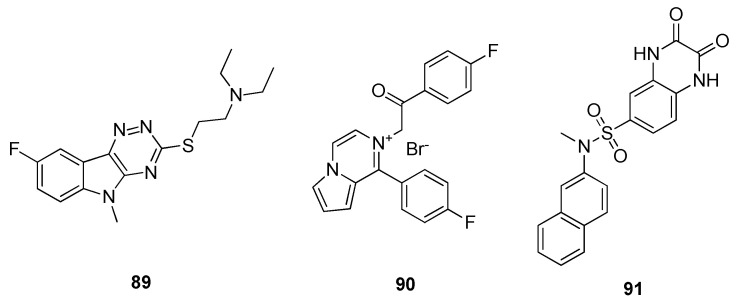
Compounds **89**–**91**, active against Eis.

In a previous manuscript, Green and co-authors [38] used three compounds (**89**–**91**, Figure 40) belonging to different structural families to demonstrate how Eis mutations can influence the potency of the inhibitors.

**Figure 41:**
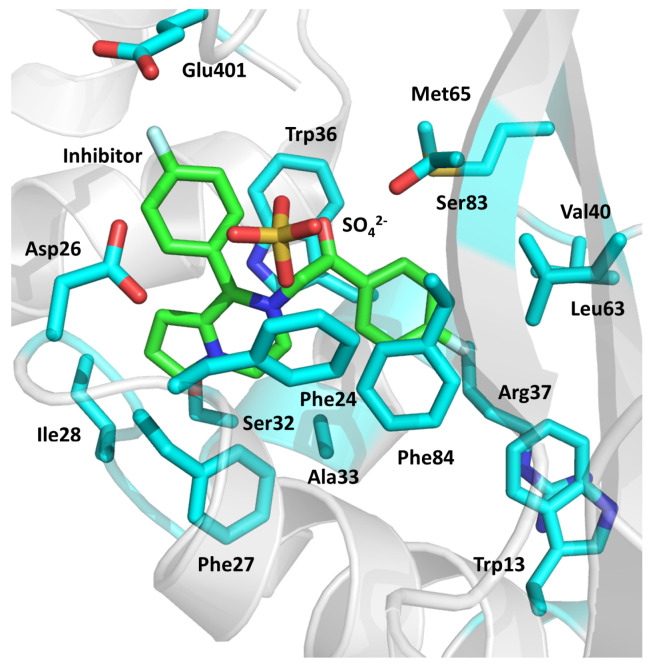
Depiction of **90** bound to Eis (PDB: 6P3U). The ligand and interacting residues are represented as green and cyan sticks, respectively.

Compound **89** belongs to the same family of analogue **87** [36] and its crystal structure showed the same interactions illustrated above for **87** and **88**. Despite belonging to a different chemical class, compound **90** was characterized by a similar interaction pattern. In detail, it established hydrophobic interactions in the binding pocket through its pyrrolopyrazine core: the pyrrole portion interacted with Ile28 and Ser32, while the pyrazine portion with Phe27 and Ala33 (Figure 41). The fluorophenyl group was positioned between Asp26 and Glu401. The cationic nitrogen of the pyrazine ring formed a cation−π contact with Trp36 and a sulfate anion, neutralizing its charge. The acetophenone ring was sandwiched between Trp36 and Phe84, and its oxygen established a weak hydrogen bond with the hydroxyl group of Ser83. Moreover, it formed contacts with a hydrophobic pocket formed by Trp13, the aliphatic stem of Arg37, Val40, Leu63, and Met65. The main interactions established by compound **91** were substantially similar to the ones reported above. The only notable difference was that its quinoxalinedione moiety formed a parallel π–π stacking interaction with the phenyl ring of Phe24. Interestingly, this residue was found to be pivotal for the acetylation of aminoglycosides, like KAN.

Starting from this information, seven residues (Asp26, Trp36, Arg37, Leu63, Met65, Ser83, and Phe84) not directly involved in the acetyl transfer function of Eis, but observed to interact with the inhibitors, were mutated into alanine residues to determine the effects on aminoglycoside acetylation and on inhibitor binding. The results showed that mutations in only three amino acids (Asp26Ala, Trp36Ala, and Phe84Ala) rendered all inhibitors inactive, suggesting that the future design of inhibitors should not depend on interactions with these residues [38].

### 2.16. 2-Succinyl-5-enolpyruvyl-6-hydroxy-3-cyclohexadiene-1-carboxylate Synthase (MenD)

**Scheme 14:**
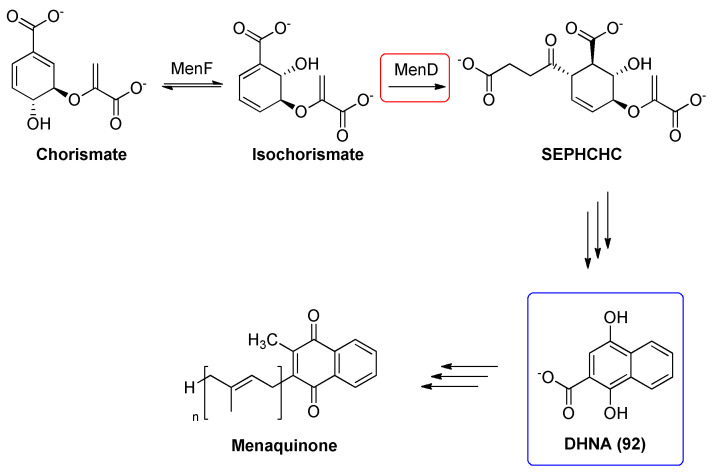
Simplified biosynthetic pathway leading to the production of menaquinone. The reaction catalyzed by MenD and the allosteric inhibitor DHNA (**92**) are highlighted with a red and blue box, respectively.

Menaquinone, also known as vitamin K_2_, is a redox cofactor consisting of a quinone core linked to a “side chain”, formed by a variable number of repeating isoprene units. This molecule is essential for energy generation in both actively growing and persistent *Mtb*, and its inhibition has been correlated to a reduced growth of the pathogen. The first step of its biosynthetic pathway is catalyzed by the thiamine diphosphate (ThDP)-dependent enzyme MenD, a member of the pyruvate oxidase (POX) family (Scheme 14). This tetrameric enzyme converts isochorismate to 2-succinyl-5-enolpyruvyl-6-hydroxy-3-cyclohexene-1-carboxylic-acid (SEPHCHC), ultimately leading to the key precursor 1,4-dihydroxy-2-naphthoic acid (DHNA, **92**) [39].

**Figure 42:**
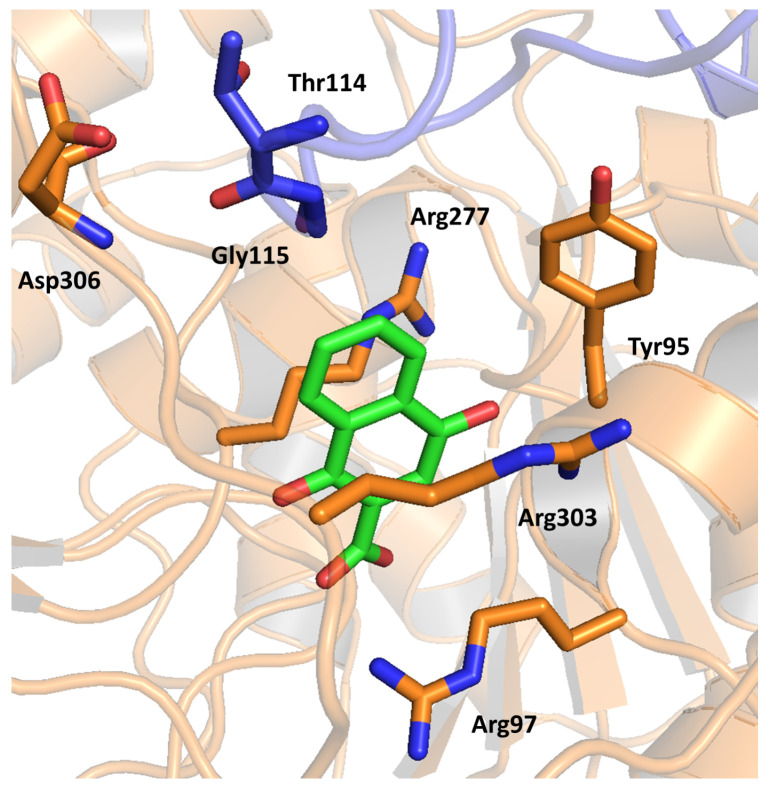
Representation of **92** (green sticks) bound to the allosteric site of MenD (PDB: 6O0N). Thr114 and Gly115, belonging to the flexible loop of the neighboring subunit, are depicted as blue sticks, while the remaining interacting residues are in orange.

Each MenD monomer is composed of three domains: while domains I and III are involved in the catalytic function, domain II does not participate directly in the binding of substrates or cofactors. Recently, Bashiri and collaborators determined that this structure is involved in the allosteric regulation of the enzyme activity. In detail, they discovered through crystallographic studies that domain II binds DHNA (**92**), a downstream intermediate of menaquinone synthesis, resulting in a negative feedback regulation. An NMR-based activity assay showed that the activity of MenD at a concentration of 5 μM was reduced by 76% in the presence of 20 μM of **92**. Further increases in the concentration of the inhibitor did not significantly influence the activity, suggesting the saturation of the enzyme. A UV spectrophotometry-based assay was employed to determine the IC_50_, which was found to be 53 nM. Because **92** acts as an allosteric inhibitor of MenD, a characterization of its binding mode may prove useful for the design of synthetic compounds capable of blocking this enzyme. The crystal structure of the MenD-**92** complex revealed that the compound bound in a cleft is located between domains I and II and formed by residues 94–97, 232–235, 276–278, 299–306, and capped by residues 112–120 from a neighboring subunit (Figure 42). In detail, the compound occupied an “arginine cage”, composed by Arg97, Arg277, and Arg303; while Arg277 and Arg303 packed on the two sides of the planar ring of **92**, Arg97 formed a H-bond with its carboxylate group. Additional H-bonds between the hydroxyl groups of the ligand and Tyr95/Arg303 contributed to stabilize the complex. The region between residues 112–120 from the other subunit of the dimer and belonging to a flexible active-site loop, interacted with **92** through van der Waals contacts with Gly115. Moreover, Thr114 bound to Asp306 of the allosteric cleft. Unsurprisingly, this mobile region was fully ordered only in the presence of the bound inhibitor. Further alanine mutagenesis experiments established the three arginine residues making up the so-called “cage” as the most important for **92** binding and feedback inhibition, indirectly corroborating their key role in signal propagation from the allosteric site to the active site of MenD [39]. Interestingly, these residues were found to be present only in bacteria closely related to *Mtb*: therefore, MenD represents an ideal, selective target for the development of novel antitubercular agents. Notably, the structural and biochemical studies established that the candidate inhibitor should have the ability to simultaneously interact with the key Arg97, Arg277, and Arg303 residues.

### 2.17. Tryptophan Synthase (TrpAB)

**Scheme 15:**
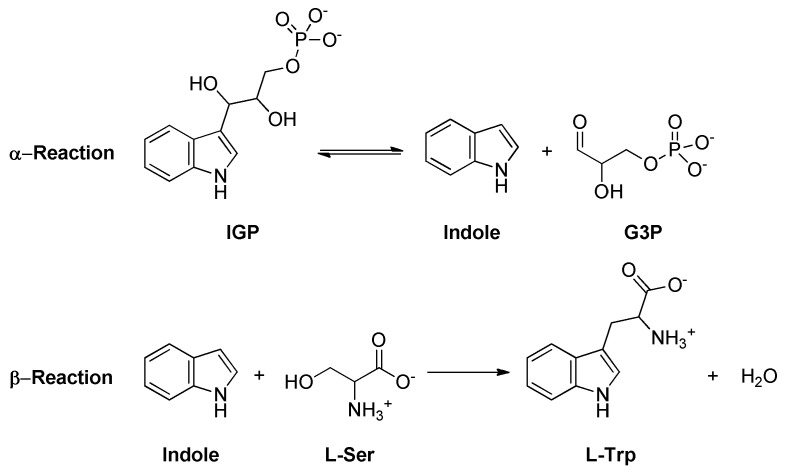
Reactions catalyzed by TrpAB, leading to the biosynthesis of l-Trp [41].

While humans and animals have lost the ability to produce tryptophan as a result of evolutionary processes, bacteria still retain the capacity to synthesize this amino acid when its exogenous concentration is low. Despite being dispensable under normal conditions, *Mtb* TrpAB becomes essential when the host adaptive immune response stimulates the expression of indoleamine 2,3-dioxygenase (IDO-1), an enzyme responsible for l-Trp degradation. TrpAB is composed of two protein subunits, α (TrpA) and β (TrpB), forming a linear αββα heterotetramer containing two active sites, separated by a 25-Å long channel. Structurally, TrpA has a (β/α)_8_-barrel fold, while TrpB is made up of two three-layer (αβα) sandwich domains. The active site of TrpA is located at the top of the central β-barrel and capped by α-Leu6. Its binding site accommodates indole 3-glycerol phosphate (IGP) and converts it to glyceraldehyde-3-phosphate and indole. The latter then travels through the channel toward the active site of TrpB, located in a cleft carrying the bound PLP cofactor, where it displaces the hydroxyl group of l-Ser to form l-Trp (Scheme 15). The activity of the enzyme is regulated allosterically by the alternation of open and closed conformations. In the open state, the active sites are accessible to the substrates; conversely, in the closed state they are sealed off, while the channel connecting the subunits remains open to allow the movement of the intermediate indole. The switch between open and closed conformations is pivotal to the activity of the enzyme and is coordinated by the so-called communication domain (COMM) [40].

**Figure 43:**
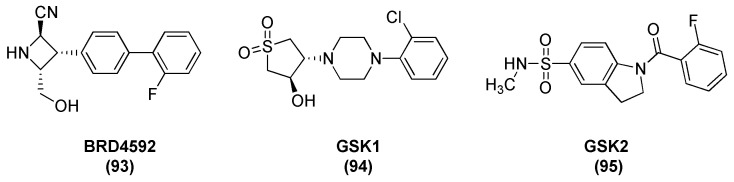
Molecular structure of the three TrpAB inhibitors.

The crystallographic structure of TrpAB was described for the first time in 2017 by Wellington and co-workers, who analyzed both the apo form and the complex with the azetidine inhibitor BRD4592 (**93**, MIC = 1.6-3 μM, Figure 43) [42]. Later, in 2020, the same group investigated the cocrystal structures of the enzyme with GSK1 (**94**, MIC = 0.76 μM *vs Mtb H37Rv*) and GSK2 (**95**, MIC = 1.1 μM *vs Mtb H37Rv*), two inhibitors developed by the University of Birmingham and GlaxoSmithKline (Figure 43) [40,43].

**Figure 44:**
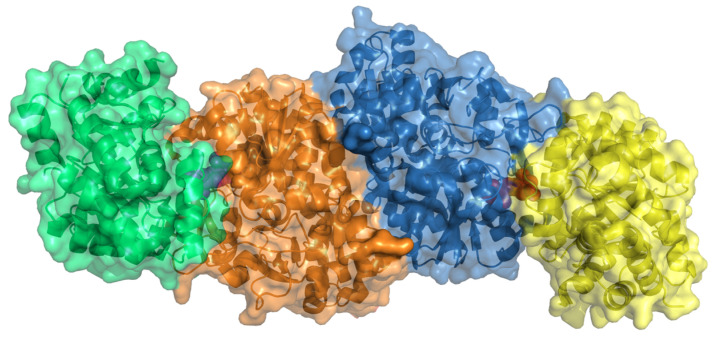
Complex of TrpAB with **94** (PDB: 6USA): subunits α are depicted in yellow and green, while subunits β are in blue and orange. **94** molecules are represented as red and magenta spheres in the allosteric binding site.

The soaking of these molecules in crystals of TrpAB (*P*2_1_2_1_2_1_) allowed the obtainment of structures at 2.4 Å resolution. The analysis of their binding mode revealed that they both bound to the same allosteric site, formed by a cavity located at the interface of the α and β subunits and intersecting the hydrophobic channel linking the two active sites. A similar binding was also observed for the previously disclosed **93**. Despite being chemically different, all three molecules share the same features, namely a large, linear hydrophobic moiety with a small, hydrophilic head. The former formed hydrophobic interactions with the side chains of β-Phe188, β-Phe202, β-Pro208, β-Ile184, and β-Leu34. The latter established contacts with different amino acids depending on the molecule, with the only exception of α-Gly66, which was shared by all three of them. In detail, **94** formed H bonds through its sulfolane moiety with the backbone amides of α-Gly66, β-His294, and α-Gly295, and with ordered water molecules (Figure 44). The sulfonamide group of **95** established H bonds with the main-chain residues α-Gly66 and α-Met67, and with the side chain of α-Asp136. Moreover, its methyl group fitted in a solvent pool located between the α and β subunits; consequently, six water molecules became highly ordered, forming stable contacts with the side chains of α-Asp136, α-Tyr62, β-Thr308, and other backbone atoms. Hence, despite **95** did not directly interact with the β subunit, its coordination network resulted in the stabilization of the α/β interface. As for **93**, the secondary amine forming the hydrophilic head interacted with α-Asp64, α-Gly66, and β-His294 [40].

Overall, the known TrpAB inhibitors stabilized the enzyme in the α^o^β^c^ form (α-open, β-closed), drastically reducing its flexibility, and hindering the communication between the α and β subunits. As a result of the obstruction of the linking tunnel, indole cannot migrate to the TrpB active site and finalize the catalytic cycle.

### 2.18. Salicylate Synthase (MbtI)

**Scheme 16:**
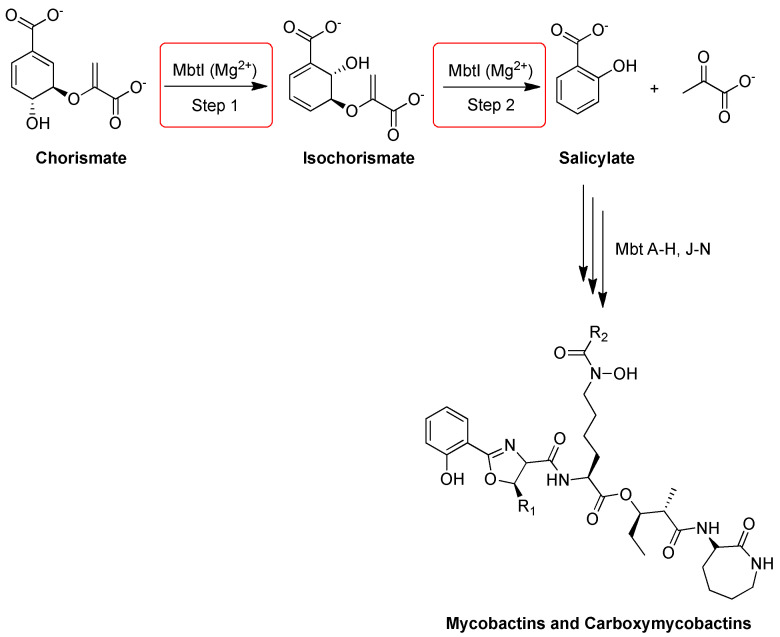
Simplified scheme of the biosynthesis of siderophores. The reactions catalyzed by MbtI are highlighted with a red box.

Iron is the essential cofactor of several biosynthetic processes involved in the survival and pathogenicity of most bacteria. Hence, the restriction of the accessibility of this metal has evolved as a fundamental part of the mammalian innate immune system. At the same time, bacteria have developed strategies to counteract this defense mechanism. *Mtb* relies on numerous pathways to internalize both heme and non-heme iron: one of the most important processes is based on the biosynthesis of iron chelators, known as mycobactins and carboxymycobactins. These low-molecular-weight siderophores have a high affinity for iron and can efficiently sequester it from host carriers. The first step of the biosynthetic process leading to the production of carboxy-/mycobactins is catalyzed by MbtI, a Mg^2+^-dependent enzyme that allows the two-step conversion of chorismate to salicylate via isochorismate (Scheme 16).

**Figure 45:**
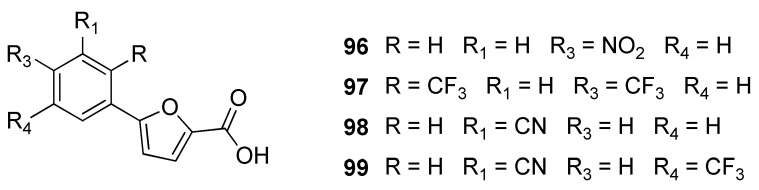
A selection of the best furan-based MbtI inhibitors.

In 2018, Chiarelli and collaborators disclosed a new class of 5-phenyl-2-furoic acid-based competitive inhibitors of MbtI, after a virtual screening campaign [44]. Subsequent SAR studies led to the discovery of more active derivatives (**96**–**99**, Figure 45), also exhibiting promising antimycobacterial effects in whole-cell assays and a negligible cytotoxicity against MRC-5 fibroblasts [45,46,47].

**Figure 46:**
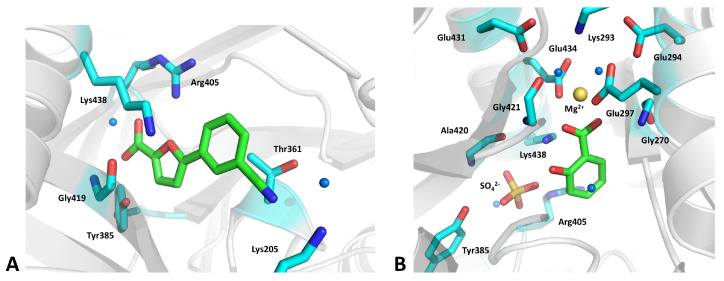
View of the binding modes of **98** (**A**) and salicylate (**B**) in the active site of MbtI. The ligands are depicted as green sticks, while the interacting residues are in cyan. The Mg^2+^ is represented as a gold sphere, while ordered water molecules are colored blue.

In 2020, Mori and collaborators successfully co-crystallized the lead inhibitor **98** (IC_50_ = 6.3 μM, MIC_99_ = 250 μM) with MbtI at a resolution of 2.09 Å (PDB: 6ZA4, Figure 46), revealing an unexpected binding mode [46]. The absence of the catalytic Mg^2+^ ion in the crystal determined the 180° rotation of the ligand with respect to the original computational simulation. Further investigations on the role of the cofactor in catalysis and inhibition revealed that MbtI has a low affinity for Mg^2+^ in its unbound form and, consequently, the Mg^2+^-ligand interaction is not necessary to block the catalytic process. The inhibitor bound to the active site, with the enzyme in open conformation. The analysis of the binding mode of the compound revealed the presence of H bonds between its carboxylic group and Tyr385, Arg405, Gly419, and an ordered water molecule; the oxygen of the furan interacted with Arg405, while the phenyl ring formed a cation−π interaction with Lys438 and a van der Waals contact with Thr361. The CN group formed a H bond with Lys205, a key amino acid involved in the first step of the catalytic reaction. Further studies on the binding of Mg^2+^ to the enzyme led to the obtainment of the first crystal structure of MbtI complexed with the ion in its physiological position (PDB: 6ZA5, Figure 46). The analysis of the electron density revealed the presence of salicylate in the active site, despite neither chorismate nor any reaction intermediate was supplied in the crystallization environment. This structure shed light on the binding mode of the substrate/product of the catalytic reaction and suggested that the enzyme was functional in the expression vector (*E. coli*). Moreover, the enzyme appeared to be in its closed conformation, having the mobile loops (268−293 and 324−336) in a fully ordered state. The Mg^2+^ ion interacted with Glu297, Glu434, and two ordered water molecules, which in turn made H bonds to Glu294 and Glu431. In addition to that, the metal was coordinated by the carboxylic moiety of salicylate, which also formed additional H bonds with the peptide backbone through Gly270 and Gly421 and with the side chain of Thr271. The hydroxyl group and the phenyl ring did not form significant interactions. The contacts between the ligand and Gly270 or Thr271 were identified as key factors influencing the enzyme conformation [46]. Overall, these structural data are being used to optimize these derivatives and may also prove pivotal to design new inhibitors of MbtI.

### 2.19. Malate Synthase G (GlcB)

**Scheme 17:**
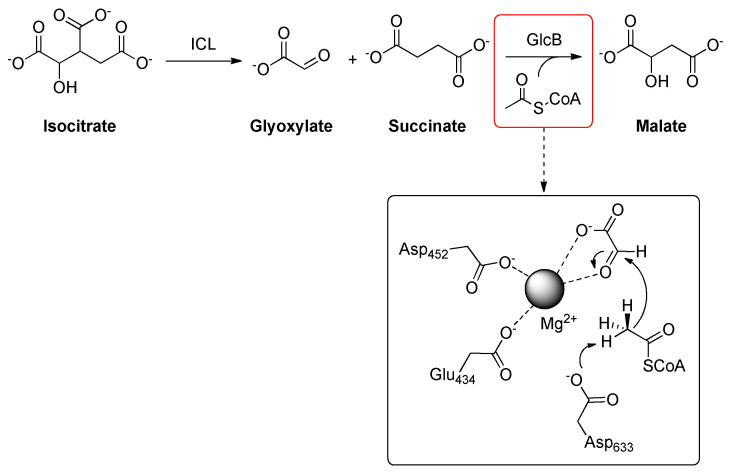
Reactions catalyzed by isocitrate lyase (ICL) and malate synthase (GlcB) [49].

GlcB is involved in the glyoxylate shunt of the tricarboxylic acid (TCA) cycle of *Mtb*. This enzyme catalyzes the condensation and subsequent hydrolysis of acetyl-coenzyme A (acetyl-CoA) and glyoxylate to form malate and CoA (Scheme 17). GlcB has been shown to play a critical role in the virulence and persistence of the mycobacterium; moreover, it has a large, accessible active site and has no human homologs [48]. Overall, these characteristics make this enzyme an ideal target for the development of anti-TB agents.

**Figure 47:**
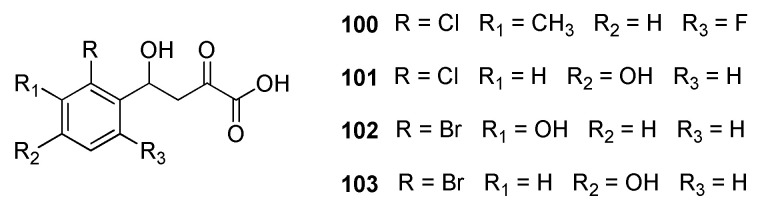
A selection of PDKA derivatives (**100**–**103**) active as GlcB inhibitors.

In 2012, Krieger and co-workers identified a new class of phenyl-diketo acids (PDKA) that inhibited GlcB by mimicking the glyoxylate substrate [50]. These structures (**100**–**103**, Figure 47) proved to be potent and non-toxic, prompting structural studies to characterize their binding mode.

**Figure 48:**
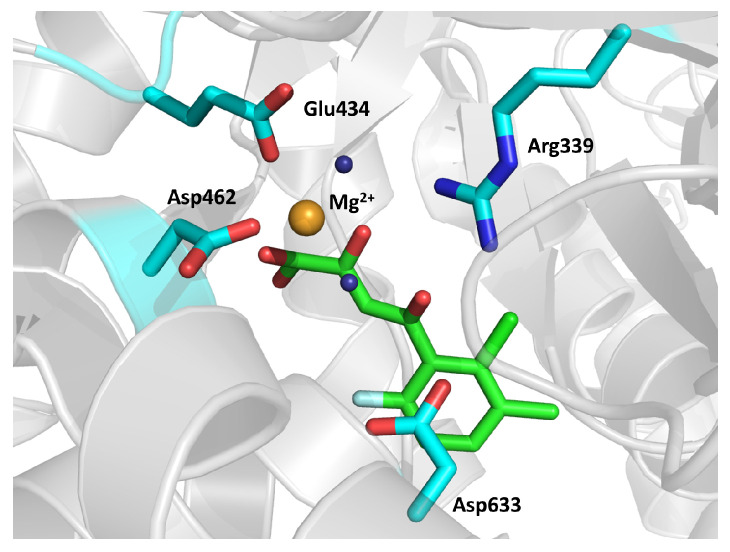
Binding mode of **100** (green sticks) in the GlcB binding site (PDB: 3SB0). Interacting residues are depicted as cyan sticks, while the Mg^2+^ ion and the water molecule are represented as gold and blue spheres, respectively.

Six years later, Ellenbarger and collaborators published twenty cocrystal structures of GlcB with different PDKA derivatives [48]. Their interaction in the active site was found to be quite conserved (Figure 48).

In detail, the ligand coordinated the catalytic Mg^2+^ cofactor with its ketoacid moiety; the octahedral sphere was completed by Glu434, Asp462, and two water molecules, which were stabilized by further contacts with the acidic residues of the active site. The positively charged Arg339 formed H bonds with the two ketones of the PDKA tail. Finally, Asp633 was involved in long-range electrostatic interactions with Arg339 and the Mg^2+^ ion, and in an anion-π contact with the phenyl group of the inhibitor, with a mean distance of ~3.5 Å. This latter interaction proved to be extremely important for the activity, but also quite sensitive to changes in the substituents around the phenyl ring. While most contact points remained relatively fixed in all the structures, the orientation of Asp633 was found to be considerably variable, impacting the formation of the interaction with the ligand. Despite a clear relationship between the substitution of PDKAs and the inhibitory activity could not be established, the authors observed that bulky or numerous, strongly electronegative substituents were linked to high IC_50_ values, whereas one or two electronegative substituents at positions 2 and 6 and a hydroxyl or methyl group at position 3 or 4 resulted in a lower IC_50_. After realizing that traditional approaches invariably led to unreliable predictions, Ellenbarger and co-workers developed an ad hoc computational model based on the crystallographic information (PDB: 3SB0, Figure 48), eventually succeeding in obtaining dependable results [48].

### 2.20. β-ketoacyl-AcpM Synthase (KasA)

**Scheme 18:**
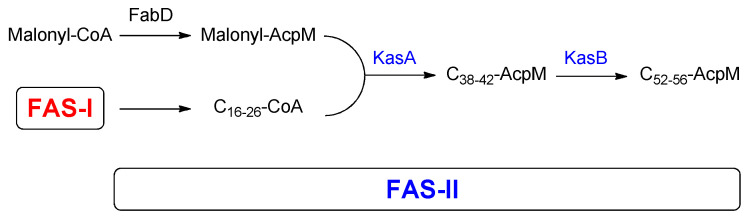
Scheme of the biosynthetic process leading to the production of meromycolic acids [52].

Cell wall biosynthesis constitutes an invaluable source of molecular targets for the development of anti-TB agents. Among its most important components are mycolic acids, C_60_-C_90_ branched-chain β-hydroxylated fatty acids that comprise the mycolyl-arabinogalactan peptidoglycan (m-AGP) cell wall complex. Their production depends on two fatty acid synthases, FAS-I and FAS-II (Scheme 18), which catalyze the elongation of fatty acid chains thus originating two branches, the carboxylated α-alkyl C_26_ fatty acid branch (FAS-I) and the meromycolic acid branch (FAS-II), which are then condensed by type I polyketide synthase 13 (Pks13). The enzymes KasA and KasB, two components of the FAS-II pathway, function in tandem to carry out acyl chain elongation to achieve meromycolic acids from acyl primers provided by FAS-I. In particular, KasA has been shown to sustain the virulence and persistence of *Mtb* in vivo, thus representing an ideal candidate for medicinal chemistry efforts [51].

**Figure 49:**
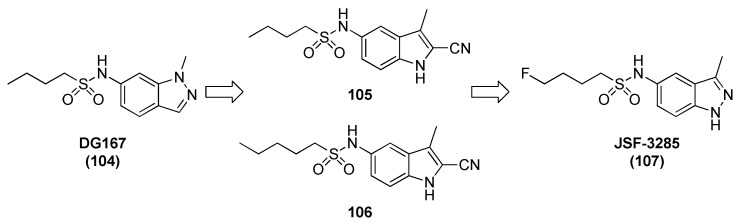
Evolution of **104**-derived KasA inhibitors.

In 2018, Kumar and collaborators reported an indazole sulfonamide inhibitor, DG167 (**104**, Figure 49), which displayed an excellent MIC of 0.39 μM against *Mtb* H37Rv and a lack of cross-resistance with first-line drugs [53]. The same compound had already been published independently by Abrahams in 2016 [54]. However, **104** proved to be inactive in vivo, likely due to pharmacokinetic issues related to its *N*-demethylation [51]. The compound was successfully crystallized with KasA at 2.0 Å (PDB: 5W2P). Despite two molecules were present in the active site, only one of them was found to be biologically relevant. It bound to the phospholipid (PL) binding site, forming hydrophobic interactions throughout the acyl channel, and establishing H bonds with Glu199 through its sulfonamide moiety [53]. In 2020, the same group designed a series of transposed indole derivatives to overcome the issues of **104** [51].

**Figure 50:**
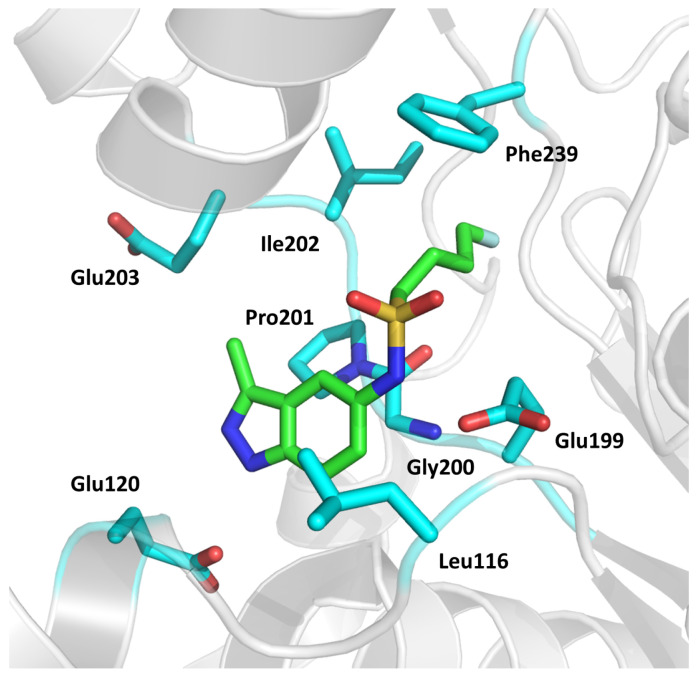
View of the binding mode of **107** within KasA (PDB: 6P9L). The ligand and interacting residues are represented as green and cyan sticks, respectively.

Crystallographic investigations around two members of this series (**105** and **106**, PDB: 6P9K and 6P9M, respectively; Figure 49) revealed that the two compounds bound to the same PL site. Their aliphatic moiety mimicked the PL acyl tail, inserting into a hydrophobic pocket; moreover, the sulfonamide and the indole N-H groups established H-bonds with Glu199 and Glu120. The two compounds differed in the positioning of the moieties and in some hydrophobic contacts. Despite the promising results in vitro, the indole series was inactive in vivo, which prompted the authors to design a new class of transposed indazole derivatives. Among them, JSF-3285 (**107**, Figure 49) emerged for its excellent antitubercular activity (MIC = 0.20 μM against *Mtb* H37Rv), lack of toxicity, and favorable pharmacokinetic profile. The crystallographic analysis (PDB: 6P9L) showed a similar binding mode with respect to the previous analogues, with the exception that the fluorobutyl chain extended deeper in the hydrophobic pocket, forming an additional interaction with Ile202 (Figure 50) [51].

Unfortunately, in the same year, Cunningham and collaborators discovered that all **104** analogues were positive to the Ames assay, revealing their mutagenic potential, linked to the formation of reactive anilines [55]. Extensive efforts were put into the modification of the scaffold. Briefly, the sulfonamide group and the aliphatic chain proved to be essential, and all changes to the nature and substitution of the six- and five-membered rings resulted in compounds that were either inactive or still mutagenic [55]. Therefore, despite the preliminary results for these compounds were extremely promising, new scaffolds for the inhibition of KasA should be investigated.

### 2.21. Dethiobiotin Synthase (DTBS)

**Scheme 19:**
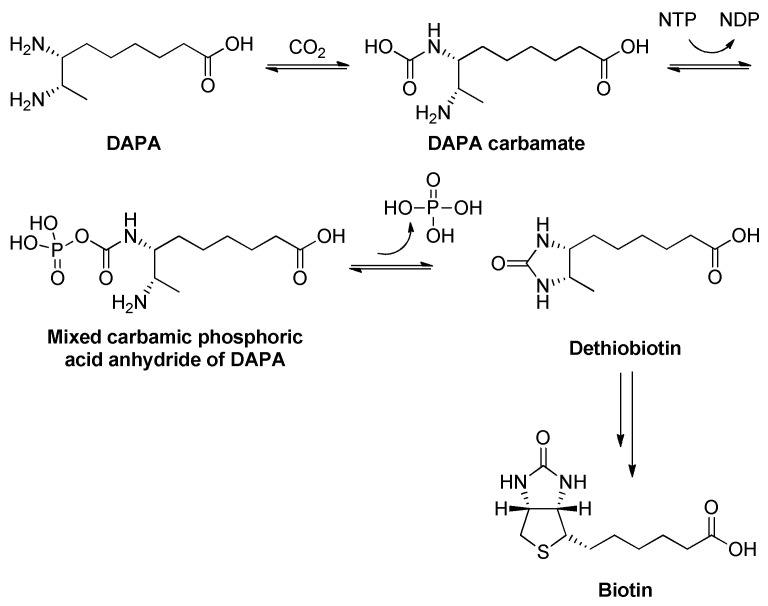
Scheme of the DTBS-catalyzed conversion of 7,8-diaminopelargonic acid (DAPA) to dethiobiotin.

Dethiobiotin synthase (DTBS) is a crucial enzyme involved in the penultimate step of the biosynthesis of biotin (Scheme 19). The inhibition of this mycobacterium-specific enzyme is an interesting strategy to fight TB due to the essential role of biotin in the cell wall lipid synthesis, during the latent phase of the life cycle of the mycobacterium.

Structural studies on DTBS showed that the enzyme was organized in biologically relevant homodimers, and that the cavity formed between two neighboring subunits hosted the DAPA binding site. The DAPA alkyl chain established hydrogen bonds through the terminal amines and the carboxylic group. Residues Gly8–Thr16 (P-loop) interacted with the basic phosphate units of the nucleotide substrate, which was also stabilized by hydrogen bonds with Gly169, Pro197, Ala200, and Ala201 (PDB: 6CVE) [56].

**Figure 51:**
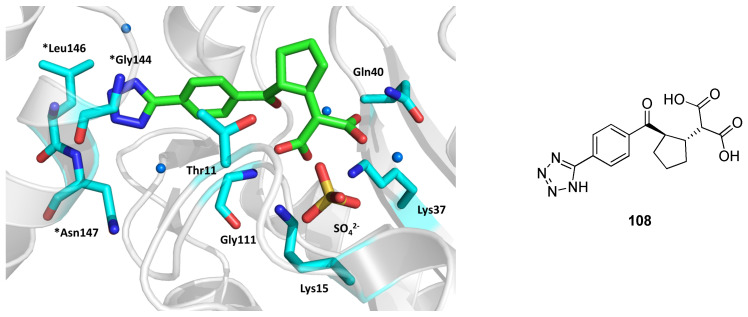
Three-dimensional view of **108** (green sticks) bound to DTBS (PDB: 7L1J). Interacting residues are represented as cyan sticks; the asterisk in the label indicates amino acids from a nearby chain. Water molecules are depicted as blue spheres.

Guided SAR studies, starting from a cyclopentylacetic acid fragment identified via an in silico screening performed by Abell et al. [57], led to the conclusion that a β-ketoacid group appended to the cyclopentyl ring and an acidic moiety at the *para* position of the aromatic ring significantly improved the binding affinity due to the formation of a critical salt bridge interaction with Lys37. Additionally, the insertion of a tetrazole on the aromatic ring, such as in **108** (*K_i_* = 5 μM, Figure 51), improved the binding affinity by around 3 orders of magnitude relative to the other binders, despite they all adopted an almost identical binding pose in the crystal structure. These studies highlighted that the tetrazole derivatives could be conveniently developed into novel antitubercular agents in future years.

## 3. Conclusions

The latest research works devoted to the structural analysis of complexes between mycobacterial enzymes and new anti-TB agents are here reported. These studies have greatly contributed to expanding the knowledge of the role of the selected targets in TB infection, as well as to identifying and developing optimized leads, also providing insights into their mechanism of action. One of the major goals of the structural biology community has always been to characterize the structures of ligand–target complexes to support the development of new drugs. As shown here, the structural data are instrumental for the hit-to-lead stage and can be successfully implemented by conventional structure-based drug design or fragment-based approaches.

Nowadays, one of the main challenges in anti-TB drug discovery is represented by the need to clarify the complex interplay among enzymes tuning *Mtb* physiology and regulating key processes in pathogenesis. In this context, the recent developments in X-ray crystallography [58], cryo-EM [59], and integrative structural biology methods [60] have contributed to increase the number of available tools to tackle these challenges. For instance, combining crystallographic and cryo-EM data may facilitate the targeting of flexible complexes and the identification of new enzymatic conformations that could not, otherwise, be evidenced by conventional structural methods. Moreover, the integration of biophysical and structural biology data, allowing the study of the targets in their larger biological context (e.g., complexes or cellular compartments), will aid the optimization of the affinity and selectivity of the enzymatic inhibitors. Taken together, these analyses will hopefully contribute to shed light on the physiology of *Mtb* and allow the design of improved anti-TB compounds.

Therefore, it is foreseeable that, despite the technical challenges, target-based and structure-based approaches will have an increasing relevance in the future of drug discovery. In this context, the recent advancements in -omics technologies have already led to the successful development of preclinical TB drug candidates, and to a better understanding of the pathogenesis of *Mtb* infection. Another fundamental tool that is going to support future research efforts is represented by the advances in pre-clinical microbiological studies that will optimize and expedite the later phases of the drug discovery process.
